# Mitochondrial dysfunction underlies cell cycle arrest and tubular hypoplasia in ClC-Kb–deficient Bartter syndrome mice

**DOI:** 10.1172/jci.insight.201067

**Published:** 2026-08-20

**Authors:** Chiao-Hui Hsieh, Yu-Jen Chen, Chih-Chien Sung, Emily Morrison, Chou-Long Huang, Chih-Jen Cheng

**Affiliations:** 1 Division of Nephrology, Department of Internal Medicine, Carver College of Medicine, University of Iowa, Iowa City, Iowa, USA.; 2 Division of Nephrology, Department of Internal Medicine, Taichung Veterans General Hospital, Taichung, Taiwan.; 3 Division of Nephrology, Department of Medicine, Tri-Service General Hospital, National Defense Medical University, Taipei, Taiwan.

**Keywords:** Cell biology, Nephrology, Cell cycle, Epithelial transport of ions and water, Mitochondria

## Abstract

The pathogenesis of Bartter syndrome (BS) has long been attributed to decreased salt reabsorption in the thick ascending limb of Henle’s loop (TAL). By studying Clc-k2–KO (mouse ortholog of ClC-Kb–KO [Clc-k2^–/–^]) mice, we recently uncovered an additional mechanism in which loss of Clc-k2 induces TAL hypoplasia in neonatal kidneys, exacerbating BS severity. Here, we further investigated this mechanism. TALs and distal convoluted tubules (DCTs) isolated from Clc-k2^–/–^ and WT mice were used for transcriptome, proteomics, cell cycle, and proliferation assays. Mitochondrial morphology and function were studied using electron microscopy and mitochondrial respiration assays. Our results reveal impairments in cell proliferation, S-to-G2/M cell cycle transition, mitochondrial biogenesis, oxidative phosphorylation, glycolysis, and fatty acid oxidation in Clc-k2^–/–^ TALs and DCTs. Increasing transport function by introducing a gain-of-function with-no-lysine kinase 4 (Wnk4) mutation in Clc-k2^–/–^ mice restored these metabolic and proliferative impairments and improved phenotype. Transgenic expression of peroxisome proliferator-activated receptor γ coactivator-1α, a master regulator of mitochondrial biogenesis, in Clc-k2^–/–^ mice also alleviated mitochondrial dysfunction and phenotype. These findings support the hypothesis that mitochondrial hypofunction, resulting from decreased transport function, contributes to cell cycle arrest and tubular hypoplasia in BS. Targeting mitochondria early in life could be a therapeutic approach for BS.

## Abstract 2

Chloride channel loss reprograms cellular and mitochondrial metabolism in kidney tubules, stalling growth; restoring transport or metabolism rescues these defects, suggesting new Bartter syndrome treatments.

## Graphical abstract

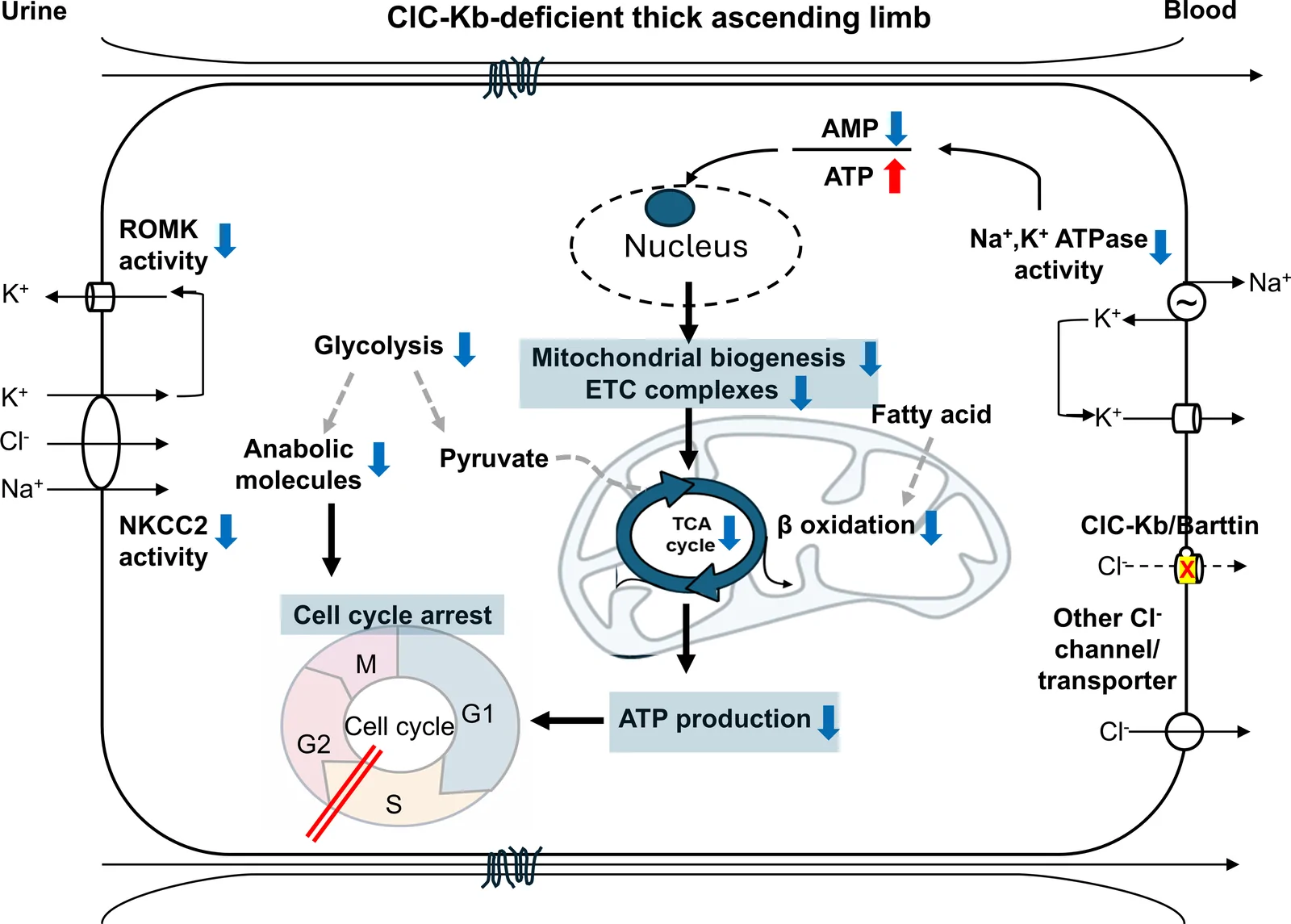

## Introduction

The voltage-gated chloride (Cl^–^) ClC-Kb channel is highly expressed on the basolateral membrane of the thick ascending limb (TAL) and distal convoluted tubule (DCT) (1). Autosomal recessive mutations in the *CLCNKB* gene result in a deficiency of the ClC-Kb channel, causing Bartter syndrome (BS) type 3 (2). The prevailing view of BS type 3 pathogenesis is that mutations disrupt transepithelial salt transport, leading to excessive renal salt wasting, urine concentration defects, secondary hyperaldosteronism, and hypokalemic alkalosis. Patients with BS type 3 commonly experience symptoms like polyuria, susceptibility to dehydration, and growth delay (3).

To explore the pathogenesis of BS type 3, we previously generated Clc-k2–KO (mouse ortholog of ClC-Kb–KO [Clc-k2^–/–^]) mice. Our findings indicated that germline Clc-k2 deletion not only impairs transport activity but also disrupts the development of TALs, as demonstrated by 3D whole-kidney imaging of 1-week-old mouse kidneys (4). In contrast, deletion of Clc-k2 induced at approximately 3 weeks of age, when mouse kidney maturation is complete, leads to a substantially milder phenotype. These results indicate that developmental defects in TALs (and likely in DCTs, given the known expression of Clc-k2 in those segments) may contribute to the pathogenesis of BS in Clc-k2^–/–^ mice. However, the mechanisms underlying the tubular hypoplasia induced by Clc-k2 deficiency remain unclear.

The TAL is the most actively proliferating segment in developing nephrons (5, 6). The elongation of TALs is critical for the maturation of the renal medulla and for maximal kidney concentrating capacity. This process requires precise regulation of cell proliferation and division with proper orientation (7). While mutations in regulators of the cell cycle, cell division, or apoptosis have been linked to diseases associated with renal medulla hypoplasia, it remains unclear whether Clc-k2 deficiency specifically disrupts these regulatory pathways in TALs (8, 9). Intracellular Cl^–^ concentration ([Cl^–^]_i_) is known to play a role in regulating the cell cycle and apoptosis (10). Whether Clc-k2 deficiency influences the cell cycle and proliferation of TALs and DCTs by altering [Cl^–^]_i_ is unclear.

Alternatively, or additionally, transport-driven changes in cell metabolism may play a role. During cell proliferation, processes such as anabolic biomass accumulation and DNA replication demand considerable metabolic support, primarily supplied by mitochondria (11). Studies across various cell types have shown that deficiencies or inhibition of the electron transport chain (ETC) complexes directly disrupt cell proliferation (12–14). Given that TALs and DCTs possess the highest mitochondrial density among all nephron segments to support active transport, they may be particularly vulnerable to metabolic shifts (15). Likewise, congenital mitochondrial disorders have been associated with TAL and DCT tubulopathies, BS, and Gitelman syndrome (16). We recently demonstrated that chronic alterations in transport activity induce reciprocal mitochondrial adaptations to maintain energy balance — a process involving the tight coupling of mitochondrial biogenesis and transport workload (17). Accordingly, we hypothesize that chronically suppressed transport activity in Clc-k2^–/–^ renal tubules triggers downregulation of mitochondrial metabolism, which in turn stalls cell cycle progression and tubular cell proliferation.

In this study, we employed a multi-omics approach combined with in vitro cell models and in vivo genetic manipulation to define the cellular consequences of Clc-k2 deficiency. We specifically investigated the interplay between transport activity, mitochondrial bioenergetics, and cell cycle progression in the TAL and DCT. By utilizing gain-of-function (GOF) with-no-lysine kinase 4 (Wnk4) and peroxisome proliferator-activated receptor γ coactivator 1-α (Pgc1α) transgenic models to restore tubular transport and mitochondrial metabolism, we demonstrate that impaired transport activity and the resulting mitochondrial hypofunction underlie the disease mechanism of BS type 3.

## Results

### Clc-k2 deficiency induces differential regulation of pathways involving cell division, proliferation, apoptosis, and mitochondrial metabolism.

To understand the cellular responses to Clc-k2 deficiency, we first conducted comparative transcriptomic analyses on TALs freshly isolated by microdissection from 6-week-old Clc-k2^–/–^ mice and their WT counterparts. All samples met the quality criteria, and none were excluded (Supplemental Figure 1A; supplemental material available online with this article; https://doi.org/10.1172/jci.insight.201067DS1). All collected data can be browsed or downloaded from the Gene Expression Omnibus (GEO) (accession number: GSE307980). Principal component analysis demonstrated distinct clustering of the 2 groups (Supplemental Figure 1B). The heatmap and volcano plot show that 1,334 genes were significantly dysregulated (624 upregulated and 710 downregulated) in Clc-k2^–/–^ mice compared with age- and sex-matched WT controls (Figure 1A and Supplemental Figure 1C). Ingenuity Pathway Analysis (IPA) based on the transcriptomic dataset revealed upregulated genes associated with cell cycle checkpoints, mitosis, and IL signaling (specifically IL-4, IL-13, and IL-17A) (Figure 1B). Conversely, the downregulated genes were associated with pathways related to phagosome formation, ion channel transport, WNK, STAT3, and mTORC1 signaling, as well as glycolysis (Figure 1B).

For proteomic analysis, which requires a larger amount of tissue, we utilized an antiuromodulin antibody to isolate TAL and early DCT cells. The authenticity of the isolated renal tubular cells was confirmed via qPCR and Western blotting (Supplemental Figure 2). All samples met the quality criteria, and none were excluded (Supplemental Figure 3A). The data can be browsed or downloaded from ProteomeXchange (accession no. PXD069389). Principal component analysis revealed that the data points from the 2 groups were clearly separated (Supplemental Figure 3B). The heatmap and volcano plot demonstrate the differential expression of 1,354 proteins (703 upregulated, 651 downregulated) in Clc-k2^–/–^ mice compared with WT controls (Figure 1C and Supplemental Figure 3C). Proteomic IPA analysis revealed that, compared with controls, Clc-k2^–/–^ TALs and DCTs presented upregulated proteins related to pathways governing mitochondrial dysfunction, IL signaling, apoptosis, and cell cycle control (Figure 1D). Conversely, proteins related to oxidative phosphorylation, biogenesis and assembly of mitochondrial respiratory complexes, mitochondrial protein degradation and import, the tricarboxylic acid (TCA) cycle, and fatty acid β-oxidation were downregulated (Figure 1D).

We then examined the correlation between the transcriptomic and proteomic datasets, which revealed robust concordance in fold changes (Figure 1E). The majority (78.6%) of differentially expressed genes show synchronous regulation at both the mRNA and protein levels (listed in Supplemental Table 1). Specifically, Clc-k2^–/–^ mice expressed lower levels of *Clcnkb* mRNA (Clc-k2 protein)*,* as expected, along with other proteins involved in electrolyte transport in TALs and DCTs, including *Wnk4* (Wnk4), *Slc12a1* (Nkcc2), and *Atp1a1* (ATPase α1-subunit), compared with WT controls (Figure 1F). Moreover, Clc-k2^–/–^ mice exhibited higher expression of *Ren* (renin), *Lcn2* (neutrophil gelatinase-associated lipocalin), *Mmp7* (matrix metalloproteinase-7), and *Cldn4* (claudin-4) (Figure 1F). These findings indicate that Clc-k2 deficiency altered salt transport and injury-related signaling pathways. Notably, some proteins were significantly downregulated in the proteomic analysis, while their mRNA levels remained relatively unchanged or were upregulated, suggesting that changes in protein expression may occur through posttranslational modifications and degradation (Supplemental Table 2). These include Ncc, Clc-ka, Barttin, and inward-rectifying potassium (Kir) channels (Kir1.1, Kir4.1, and Kir5.1). Overall, the results support the robustness of our multi-omics approach.

### Clc-k2 deficiency impairs cell proliferation and the S-to-G2/M cell cycle transition.

Postnatal TAL development requires complex regulation of cell proliferation and apoptosis to achieve tubular elongation. Our multi-omic pathway analysis confirmed that several cell cycle regulators promoting progression from the G1 to the S phase were upregulated, including *Cdk1/2/4/6* (cyclin-dependent kinases, Cdk1/2/4/6), *Ccne1/2* (cyclin E1/2), *Ccna2* (cyclin A2), and *E2f1* (E2f), whereas the cell cycle inhibitor *Cdkn1b* (p27) was downregulated (Figure 2A). In addition, both intrinsic and extrinsic apoptosis pathway–related proteins, including *Casp3/7/8/9* (Caspases 3, 7, 8, and 9), *Parp3/9* (poly [ADP‒ribose] polymerases 3 and 9), *Bax* (BCL2-associated X protein), and *Bid* (BH3 interacting-domain death agonist), were significantly upregulated (Figure 2B).

To validate the multi-omics data, we quantified proliferative tubular cells in vivo in developing TALs and DCTs. Two-week-old mouse kidneys were used for immunofluorescence costaining of Pcna (proliferating cell nuclear antigen, a marker of cell proliferation) and markers for TALs (Nkcc2) or DCTs (Ncc). Compared with control kidneys, Clc-k2^–/–^ kidneys presented significantly fewer PCNA(+) TAL and DCT cells, suggesting that these tubular cells are less proliferative (Figure 2C). To assess apoptosis, we conducted a TUNEL assay combined with immunofluorescence staining for Nkcc2 or Ncc. Compared with their WT counterparts, Clc-k2^–/–^ kidneys had more TUNEL(+) TAL and DCT cells, confirming that Clc-k2 deficiency increased apoptosis in renal tubular cells (Figure 2D). Notably, many TUNEL(+) cells were observed in non-TAL/DCT cells, including proximal tubules and collecting ducts (Supplemental Figure 4), indicating that non-cell–autonomous effects of Clc-k2 deficiency also enhance apoptosis.

To determine whether Clc-k2 deficiency directly affects the proliferation of TAL cells, we used primary TAL cells and ClC-Kb plasmid-transfected HEK-293 cells for validation. First, we confirmed the expression of Nkcc2 and Clc-k2 in primary-cultured TAL cells (Figure 3A). Compared with WT TALs, Clc-k2^–/–^ TAL cells presented no detectable Clc-k2 and reduced Nkcc2 expression. These cells were then used in CCK-8 cell proliferation assays, which measure viable cells based on the amount of formazan dye produced by dehydrogenases in living cells. The results indicate that Clc-k2^–/–^ TAL cells proliferated ~40% less efficiently than WT TAL cells did (OD 450 at Day 3, WT: 0.31 ± 0.15 versus Clc-k2^–/–^: 0.18 ± 0.12, *P* = 0.03) (Figure 3B). In addition, we applied the nonspecific Cl^–^ channel inhibitor 5-nitro-2-(3-phenylpropylamino) benzoic acid (NPPB) to HEK-293 cells transfected with ClC-Kb/Barttin or empty vector plasmids. NPPB inhibited the transition from S to G2/M phase in ClC-Kb/Barttin plasmid-transfected HEK-293 cells, causing fewer cells to progress to the G2/M phase (DMSO: 13.4% ± 6.2% versus NPPB: 4.1% ± 3.0%; *P* < 0.05) (Figure 3C), consistent with assay validation using the known cell cycle modulator H_2_O_2_ (Supplemental Figure 5). Consequently, more cells stayed at the S phase (DMSO: 41.5% ± 5.4% versus NPPB: 51.1% ± 4.6%; *P* = 0.03). The effect of NPPB was less pronounced but still significant in empty vector-transfected HEK-293 cells (G2/M phase, DMSO: 10.0% ± 2.8% versus NPPB: 6.3% ± 1.1% [*P* = 0.03]; S phase, DMSO: 42.2% ± 5.2% versus NPPB: 48.7% ± 1.8% [*P* = 0.04]). These findings suggest that Clc-k2 is critical for the S to G2/M transition.

### Reduced mitochondrial mass and oxidative phosphorylation in Clc-k2–deficient TALs.

Pathways regulating mitochondrial biogenesis and oxidative phosphorylation are among the top-regulated pathways in Clc-k2^–/–^ TALs and DCTs. Figure 4 illustrates the coordinated downregulation of multiple genes and proteins that play crucial roles in mitochondrial oxidative phosphorylation. These proteins include those that assemble electron transport complexes I, II, III, IV, and V, as well as pyruvate dehydrogenase and all key enzymes in the TCA cycle, including citrate synthase, aconitase, isocitrate dehydrogenase, α-ketoglutarate dehydrogenase, succinyl-CoA synthetase, succinate dehydrogenase, and malate dehydrogenase. Additionally, we observed a consistent downregulation of enzymes responsible for glycolysis (including hexokinase 1, phosphofructokinase, aldolase, phosphoglycerate kinase, phosphoglycerate mutase 2, enolase, and pyruvate kinase) and enzymes involved in the transport or oxidation of fatty acids, such as carnitine palmitoyltransferase 1 and 2 (CPT1 and 2), acyl-CoA dehydrogenase, enoyl-CoA hydratase, hydroxyacyl-CoA dehydrogenase, and thiolase, in Clc-k2^–/–^ TALs and DCTs (Figure 5).

We next examined mitochondrial morphology in the TALs using transmission electron microscopy (TEM). Compared with WT TALs, Clc-k2^–/–^ TALs exhibited significantly lower mitochondrial density (WT: 49.4 ± 8.5 versus Clc-k2^–/–^: 35.9 ± 4.4, *P* = 0.0003), crista density (WT: 1.45 ± 0.36 versus Clc-k2^–/–^: 1.14 ± 0.28, *P* < 0.0001), and length (Figure 6A). In addition, the mitochondrial DNA content in Clc-k2^–/–^ TALs was approximately 50% of that in WT controls (WT: 1.03 ± 0.11 versus Clc-k2^–/–^: 0.48 ± 0.19, *P* = 0.0003) (Figure 6B). Furthermore, we performed Seahorse flux analysis to measure the oxygen consumption rate (OCR) and extracellular acidification rate (ECAR) in freshly isolated TALs. Multiple mitochondrial respiratory parameters, including basal respiration, ATP-linked respiration, and maximal respiratory capacity, were significantly decreased in Clc-k2^–/–^ TALs (Figure 6C). These results confirm that Clc-k2 deficiency significantly reduces both mitochondrial mass and respiration in the TALs.

### Wnk4 restores transport function and rescues the phenotype and cellular effects of Clc-k2 deficiency.

WNK4 kinase is a master activator of salt reabsorption in the DCTs, and likely in the TALs, by phosphorylating and activating Ncc and Nkcc2. The WNK4 GOF D561A mutation is linked to pseudohypoaldosteronism type 2, a congenital thiazide-responsive hypertensive disorder in humans. Mice carrying this mutation exhibit increased transport in the TALs and DCTs, along with elevated levels of phosphorylated Nkcc2 and Ncc (18). Moreover, we previously observed an increase in mitochondrial mass and respiration in isolated Wnk4^D561A/+^ DCTs, suggesting that GOF Wnk4 may rescue the cellular effects of Clc-k2 deficiency (17). To determine whether Wnk4^D561A/+^ rescues BS type 3, we crossed Clc-k2^–/–^ mice with Wnk4^D561A/+^–knock-in mice and compared their phenotype to that of Clc-k2^–/–^ mice without or with salt supplementation, the standard treatment for BS.

Both Wnk4^D561A/+^ and salt supplementation prevented neonatal death and improved blood pressure and biochemical abnormalities, including renal function, alkalosis, and hyperreninemia; however, hypokalemia remained unchanged (Figure 7A and Table 1). Notably, Wnk4^D561A/+^ was more effective than salt supplementation at improving hypochloremia, hyperreninemia, polyuria, and renal salt wasting. In contrast, salt supplementation dramatically exaggerated urine output (~2.5-fold) and renal salt excretion (~14-fold). Additionally, the Wnk4 D561A mutation alleviated the growth delay of Clc-k2^–/–^ neonates, whereas salt supplementation did not (Figure 7B). In a 24-hour water deprivation test, Clc-k2^–/–^/Wnk4^D561A/+^ mice exhibited higher kidney concentrating capacity, less weight loss, and better responsiveness to DDAVP than Clc-k2^–/–^ mice (Figure 7C). At the molecular level, Clc-k2^–/–^/Wnk4^D561A/+^ mice had significantly higher levels of phosphorylated Ncc and phosphorylated Nkcc2 proteins than Clc-k2^–/–^ mice (Figure 7D). These findings are consistent with the stronger diuretic response observed in Clc-k2^–/–^/Wnk4^D561A/+^ mice (thiazide-sensitive urine Na^+^ excretion: Clc-k2^–/–^: 4.9 ± 3.6; Clc-k2^–/–^/Wnk4^D561A/+^: 19.2 ± 7.0 μmol/hr, *P* < 0.0001) (furosemide-sensitive urine Na^+^ excretion: Clc-k2^–/–^: 3.1 ± 4.2; Clc-k2^–/–^/Wnk4^D561A/+^: 15.5 ± 11.2 μmol/hr, *P* = 0.001) (Figure 7E).

Microscopically, adult Clc-k2^–/–^ kidneys exhibited extensive tubulointerstitial fibrosis, juxtaglomerular (JG) apparatus hyperplasia, and diffuse macrophage infiltration in the renal interstitium, consistent with the pathological hallmarks of BS (Figure 8). While salt supplementation improved JG apparatus hyperplasia, it did not reduce interstitial fibrosis and actually exacerbated macrophage infiltration. Importantly, the Wnk4 D561A mutation significantly alleviated all histological abnormalities associated with BS in Clc-k2^–/–^ mouse kidneys.

Furthermore, we isolated and cultured TALs from Clc-k2^–/–^/Wnk4^D561A/+^ mice for cell proliferation and cell cycle assays. Compared with WT TALs, Clc-k2^–/–^/Wnk4^D561A/+^ TALs presented undetectable Clc-k2 and reduced Nkcc2, similar to Clc-k2^–/–^ TALs (Figure 9A). However, these cells proliferated only ~25% less efficiently than WT TALs (OD 450 at Day 3: WT: 0.28 ± 0.06 versus Clc-k2^–/–^/Wnk4^D561A/+^: 0.21 ± 0.07, *P* = 0.04), which was better than the ~40% reduction observed in unrescued Clc-k2^–/–^ TALs (Figure 9B). In addition, cell cycle analysis revealed a marked reduction in the percentage of cells in G2/M phase in Clc-k2^–/–^ TALs compared with WT, which was largely restored in Clc-k2^–/–^/Wnk4^D561A/+^ TALs (percentage of cells in G2/M phase: WT: 21.1% ± 1.0%, Clc-k2^–/–^: 4.7% ± 1.3%, Clc-k2^–/–^/Wnk4^D561A/+^: 17.9% ± 3.1%; WT versus Clc-k2^–/–^, *P* = 0.0003; Clc-k2^–/–^ versus lc-k2^–/–^/Wnk4^D561A/+^, *P* = 0.018; WT versus Clc-k2^–/–^/Wnk4^D561A/+^, *P* = 0.37) (Figure 9C). Compared with Clc-k2^–/–^ mice, Clc-k2^–/–^/Wnk4^D561A/+^ mice showed significant improvements in mitochondrial morphology in the TALs, whereas salt supplementation did not (Supplemental Figure 6). Principal component analysis of transcriptomic data revealed that Clc-k2^–/–^/Wnk4^D561A/+^ showed high similarity to WT TALs and clear separation from Clc-k2^–/–^ TALs (Figure 9D). The heatmap further shows that Wnk4^D561A/+^ partially corrects the differentially regulated genes in Clc-k2^–/–^ TALs (Figure 9E). These findings suggest that GOF Wnk4 improves the cellular consequences of Clc-k2 deficiency.

### Pgc1α improves the phenotype of Clc-k2^–/–^ mice.

Pgc1α is a master activator of mitochondrial biogenesis and bioenergetics. To determine whether Pgc1α can rescue the BS phenotype in Clc-k2^–/–^ mice, we crossed Pax8-rtTA/LC1/*Clcnkb*^fl/fl^ mice with Pgc1α transgenic (Pgc1αTg) mice and induced Clc-k2 deletion and Pgc1α transgene expression by feeding doxycycline during pregnancy. Compared with noninducible (vehicle-treated) controls, Clc-k2^–/–^/Pgc1α(–) mice expressed significantly less Pgc1 protein, whereas Clc-k2^–/–^/Pgc1α(+) mice expressed a comparable amount of Pgc1 protein (Figure 10A). Both Clc-k2^–/–^/Pgc1α(–) and Clc-k2^–/–^/Pgc1α(+) mice had residual Clc-k2 protein levels of less than 3%.

Pgc1α transgene expression significantly improved mitochondrial morphology and mitochondrial DNA copy number in inducible Clc-k2^–/–^ kidneys; it also improved mitochondrial respiration capacity in isolated Clc-k2^–/–^ TALs (Figure 10, B and C, and Supplemental Figure 7). Phenotypically, the Pgc1α transgene ameliorated azotemia, hypochloremia, metabolic alkalosis, and renal salt wasting in inducible Clc-k2^–/–^ mice (Table 2). Furthermore, Pgc1α transgene expression partially restored the activity of Nkcc2 and Ncc in these mice, as indicated by elevated levels of phosphorylated Nkcc2 and Ncc proteins and increased diuretic-sensitive urine Na^+^ excretion (Figure 10, D and E). In addition, the Pgc1α transgene improved interstitial fibrosis in inducible Clc-k2^–/–^ kidneys (Supplemental Figure 8).

## Discussion

Loss of transport function has long been the central dogma of BS pathogenesis. However, our recent study using a BS type 3 mouse model revealed that germline Clc-k2 deletion results in unexpected TAL hypoplasia and medullary developmental defects via unknown mechanisms (4). Our current findings illustrate a robust crosstalk between transport function, mitochondrial metabolism, and cell proliferation. Chronic suppression of transport activity appears to reprogram tubular cell metabolism, leading to mitochondrial deficiency and cell cycle arrest.

Beyond systemic factors like hypovolemia or ischemia — secondary consequences (non-cell–autonomous effects) of Clc-k2 deficiency that can delay the G1–S transition via p21 and p27 and enhance apoptosis (19) — our in vitro data suggest that Clc-k2 deficiency directly influences cell cycle progression in the TAL and DCT. This effect is cell autonomous and particularly evident during the S to G2/M transition. Prolonged S to G2/M arrest induced by p53 leads to premature cellular senescence (20). While the exact mechanism remains unknown, Clc-k2 deficiency likely increases [Cl^–^]_i_ due to impaired efflux (21). Given that elevated [Cl^–^]_i_ is known to inhibit proliferation in other cell types, our results raise the intriguing possibility that intracellular Cl^–^ accumulation may contribute to the S to G2/M arrest and subsequent TAL hypoplasia observed in Clc-k2^–/–^ mice (22).

Alternatively, or additionally, ClC-Kb deficiency downregulates mitochondrial function, which is paramount for cell cycle progression and proliferation in various tissues (11). Previous studies have demonstrated that genetic or pharmacological inhibition of transport alters mitochondrial metabolism and cell proliferation in the TAL (17, 23). The Wnk4^D561A/+^ mutation, which stimulates salt transport in the TAL and DCT, improves Ncc and Nkcc2 activity, mitochondrial metabolism, TAL cell proliferation, and the BS phenotype in Clc-k2^–/–^ mice. These improvements support the idea that impaired transport activity is the primary driver of these mitochondrial and cellular changes. Moreover, the Pgc1α transgene, a known rescuer of mitochondrial biogenesis and bioenergetics, also improves Ncc and Nkcc2 activity and the BS phenotype in Clc-k2^–/–^ mice, although we lack a control group with Pgc1α overexpression alone without Clc-k2 deficiency. This reinforces the concept of transport-mitochondria coupling and the importance of transport dysfunction–induced mitochondrial hypofunction in the disease mechanism of BS. However, we cannot fully exclude the possibility that other transport-unrelated Wnk4- or Pgc1α-specific signaling pathways contribute to these improvements.

Common cellular adaptations in various loss-of-function renal tubules include reduced cell volume, diminished DNA synthesis and mitochondrial mass, and increased apoptosis (23–25). Changes in cell metabolism may underlie these adaptations (26, 27). While TALs and DCTs are typically mitochondria rich to support high energy demands (28), we demonstrated for the first time to our knowledge that Clc-k2 deficiency induces comprehensive metabolic reprogramming where glycolysis, fatty acid oxidation, and the TCA cycle are simultaneously suppressed. Because these pathways provide both ATP and essential biomolecules for proliferation, such severe mitochondrial dysfunction is detrimental to development (29). This is evidenced by models of primary mitochondriopathy, such as *Uqcrb* deletion or *RMND1* mutation, which result in severe renal dysgenesis (30, 31). Due to transport-mitochondria coupling, any irreversible impairment in transport or mitochondrial metabolism disrupts normal cell metabolism and likely hinders tubular cell proliferation and survival.

Additionally, our rescued mouse models provide further insights. The significant improvement in endogenous Ncc and Nkcc2 activity in Wnk4-rescued Clc-k2^–/–^ mice indicates that Clc-k2 is not the sole pathway for basolateral Cl^–^ exit in TALs and DCTs. Other Cl^–^ channels or transporters, such as K^+^ and Cl^–^ cotransporters, may compensate for the loss of Clc-k2. The commonly considered/hypothesized Clc-k1 (mouse ortholog of ClC-Ka) is unlikely to compensate, as it is downregulated in the proteomic data of Clc-k2^–/–^ TALs and DCTs. Despite phenotypic improvements, GOF Wnk4 did not ameliorate hypokalemia. The persistent renal K^+^ wasting likely results from a combination of high urine flow rate and residual secondary aldosteronism, driving K^+^ secretion via Maxi-K and ROMK channels. While the exact mechanism by which Pgc1α improves the activity of Ncc and Nkcc2 in Clc-k2^–/–^ mice remains unknown, our study provides a proof of principle for a potential BS type 3 treatment strategy. Given that human kidney maturation — especially the TAL and medulla — continues from birth through puberty, targeting mitochondrial biogenesis during early childhood may offer a window of opportunity to preserve tubular function and improve long-term outcomes (32).

In summary, our results advance the understanding of cellular responses to Clc-k2 deficiency (or, more broadly, germline TAL and DCT transport dysfunction), highlighting transport dysfunction–induced metabolic reprogramming, mitochondrial deficiency, and cell cycle delays as key factors in BS type 3 pathogenesis. Future research should further investigate crosstalk between transport and mitochondrial function as well as the potential for early therapeutic interventions targeting mitochondria during the critical stages of childhood kidney development in patients with BS.

## Methods

### Sex as a biological variable.

Both sexes were examined; no sex-based differences were observed, so data were pooled.

### Animals and balanced studies.

Kidney-specific *Clcnkb*-KO (Clc-k2^–/–^) mice were created as previously described (4). Wnk4^D561A/+^ knock-in mice were provided by Shinichi Uchida at Tokyo Medical and Dental University, Tokyo, Japan (18). These mice share a common C57BL/6JNarl background and were interbred to produce Clc-k2^–/–^/Wnk4^D561A/+^ mice. For salt supplementation, Clc-k2^–/–^ mice were given s.c. injections of 0.9% saline (60 μL/g body weight) from postnatal day 1 to 3 weeks old and then fed a high-salt diet (4% NaCl, TestDiet, 5SWH) thereafter. FVB-Tg(tetO-Ppargc1a)1Dpk/J (Pgc1αTg) mice were purchased from the Jackson Laboratory and crossed with Pax8-rtTA/LC1/*Clcnkb*^fl/fl^ mice (4). Pax8-rtTA/LC1/*Clcnkb*^fl/fl^/Pgc1αTg parental mice were given 2% sucrose drinking water containing 2 mg/dL doxycycline during pregnancy and postpartum lactation to induce embryonic *Clcnkb* deletion with or without Pgc1α expression in the offspring. All offspring continued drinking doxycycline-containing water after weaning. Mice aged 6–12 weeks were used for the experiments. Blood samples were obtained via retro-orbital bleeding, and urine samples were collected by housing the mice individually in an MMC100 metabolic cage (Hatteras Instruments). Plasma biochemistry and gas analysis results were measured with an iSTAT (Abbott). Urine biochemistries were measured with a Diamond Prolyte electrolyte analyzer (Diamond Diagnostics). Systolic and diastolic blood pressures were measured as previously described (4).

For the diuretic tests, the mice were injected i.p. with vehicle (2.5% DMSO in 0.9% NaCl) and then placed in metabolic cages for 4 hours to collect urine samples. The next day, a designated diuretic (hydrochlorothiazide 12.5 mg/kg or furosemide 15 mg/kg) was injected i.p., and a 4-hour urine sample was collected for analysis. Diuretic-sensitive sodium excretion was calculated by subtracting vehicle-induced natriuresis from diuretic-induced natriuresis. In the water deprivation test, the mice were deprived of water for 24 hours, and urine samples were collected at 0, 6, 12, and 24 hours. After a 24-hour water deprivation period, 1-deamino-8-D-arginine vasopressin (DDAVP, 0.4 ng/g body weight, Sigma) was administered i.p. Urine samples were collected again 2 hours after DDAVP injection.

### Renal tubule microdissection.

Renal tubules were microdissected as previously described (Supplemental Methods) (4). The isolated TALs were utilized for qPCR, transcriptome analysis, primary cell culture, cell cycle and proliferation assays, DNA copy number analysis, and Seahorse assays.

### Purification of mouse primary TAL and DCT cells.

To obtain sufficient cells for proteomics, we pulled down uromodulin^+^ TAL and early DCT cells as previously described (Supplemental Methods) (33).

### Transcriptome analysis.

Microdissected TAL segments were washed in 1× Dulbecco’s PBS with CaCl_2_ and MgCl_2_ under a stereomicroscope. The tubules were transferred into a 1.5 mL microcentrifuge tube containing 2 μL of Dulbecco’s PBS for total RNA extraction via the RNeasy Plus Mini Kit (Qiagen). Total RNA was eluted in 10 μL of RNase-free water. For RNA-Seq, cDNA was synthesized using a SMART-Seq V4 (Takara Bio., 634769) according to the manufacturer’s protocol. The cDNA was then amplified for 15 PCR cycles, purified with AmPure XP magnetic beads (Beckman Coulter Genomics, A63880), and eluted with 15 μL of elution buffer. The purified cDNA was quantified fluorometrically using a Qubit 2.0 fluorometer. Library size distribution was determined via an Agilent 2100 bioanalyzer with a high-sensitivity DNA kit (Agilent Genomics, 5067-4626). SMART-Seq mRNA LP was used to generate the libraries, with an input of 1 ng of cDNA per sample. Libraries were pooled and sequenced to obtain 75-base-pair paired-end reads on the Illumina NextSeq 550 platform, yielding > 20 million reads per sample. The results of the bioinformatics analysis are detailed in the Supplemental Materials.

### Proteomic analysis.

The protein concentration was determined using the Qubit Protein Assay Kit (Thermo Fisher Scientific, Q33212), and 20 μg of protein was used for downstream processing. To each sample, 0.1M tris(2-carboxyethyl)phosphine (TCEP) was added to achieve a final concentration of 5 mM, followed by alkylation with 10 mM chloroacetic acid (CAA) at 95°C for 10 minutes. Proteins were acidified with 43% phosphoric acid to a final concentration of 27.5% and then mixed with methanol/100 mM triethylammonium bicarbonate (TEAB) on ice for 5 minutes. The mixture was loaded onto S-Trap micro columns (ProtiFi LLC, C02-micro80) and centrifuged at 4,000*g* for 30 seconds; this process was repeated 3 times. The columns were washed 3 times with 90% methanol in 100 mM TEAB. Proteins were digested on columns with trypsin at 37°C for 16 hours. Peptides were eluted sequentially with 50 mM TEAB, 0.2% formic acid (FA), and 50% acetonitrile (ACN). Each sample was desalted using C18 Spin Tips (Thermo Fisher Scientific, ZJ394337). The dried peptides were resuspended in 50% trifluoroacetic acid (TFA) to adjust the pH to 2–3. C18 Tips were prewetted with 80% ACN/0.1% TFA and equilibrated with 0.1% TFA. The peptides were bound to the tips by aspirating and dispensing the solution 3 times, followed by washing with 0.1% TFA. Elution was performed using 20 μL of 70% ACN/0.1% TFA, which was also aspirated and dispensed 3 times. The eluates were centrifuged at 20,000*g* for 10 minutes, dried, and then resuspended in 3% ACN/0.1% FA for LC-MS analysis (Supplemental Methods).

### Cell culture and plasmid transfection.

Dissected TALs were transferred and cultured on 0.33 cm^2^ collagen-coated PTFE filter membranes containing DMEM:F12 (Gibco-BRL) culture medium with 0.01% nonessential amino acids, 2% FBS, and one batch of SingleQuots (Lonza, CC-4127) in a humidified chamber at 37°C and 5% CO_2_, grown into an ~70% confluent monolayer, and then switched to low-serum (0.1% FBS) medium to allow maximal differentiation. Human embryonic kidney (HEK-293) cells were cultured in DMEM (Thermo Fisher Scientific) supplemented with 10% fetal bovine serum (Thermo Fisher Scientific) and 1% penicillin-streptomycin. The cells were maintained at 37°C in a humidified incubator with 5% CO_2_. For transfection, HEK-293 cells were seeded at a density of 2 × 10^5^ cells per well in 6-well plates and incubated for 24 hours. The cells were then transfected with 1 μg total plasmid DNA (1 μg pCMV6-AC-GFP empty vector [PS100010] or 0.5 μg pCMV6-AC-ClC-Kb–GFP [RG228424] plus 0.5 μg pCMV6-AC-Barttin-GFP [RC213125]; Origene) using Lipofectamine 3000 (Thermo Fisher Scientific) in 500 μL of serum-free DMEM. After 6 hours, the transfection medium was replaced with complete growth medium containing serum.

### Cell proliferation analysis.

A Cell Counting Kit-8 (Dojindo Molecular Technologies, CK04-01) was used to measure viable cells in the cell proliferation assay by colorimetric analysis. Briefly, equal amounts of TAL cells were seeded into a 96-well plate and incubated for 24, 48, or 72 hours. For colorimetric measurement, 10 μL of Cell Counting Kit-8 reagent was added to each well. After 1.5 hours of incubation at 37°C, the absorbance of each well was measured at 450 nm.

### Cell cycle analysis.

One-week-old primary-cultured TAL cells or ClC-Kb/Barttin plasmid-transfected HEK-293 cells (ATCC, CRL-1573) were used for cell cycle analysis. For pharmacological inhibition of ClC-Kb, HEK-293 cells (4 × 10^5^ cells/well) were seeded in 6-well plates for 24 hours and then treated with 150 μM NPPB (Sigma-Aldrich, N4779) or vehicle (DMSO) for 24 hours. HEK-293 cells were also treated with 100 μM furosemide or 0.2 M H_2_O_2_ for negative and positive controls, respectively. The cells were harvested and fixed in 70% ethanol overnight at 4°C. Fixed cells were washed and resuspended in PBS containing 100 μg/mL RNase A and 0.1% Triton X-100, then stained with 5 μg/mL propidium iodide (PI) for 30 minutes at 37°C. The cell cycle distribution was analyzed using a BD LSR II flow cytometer (BD Biosciences).

### Western blot analysis, renal histology, and immunofluorescence staining.

The kidneys were harvested from the mice immediately after sacrifice for Western blot analysis and immunofluorescence (IF) exams as previously described (4). The protocol and antibodies used are detailed in the Supplemental Methods. Immunofluorescence images were obtained via confocal microscopy (Zeiss LSM 880), whereas H&E- and Masson trichrome–stained samples were scanned via Zeiss Axioscan Z1. Tubulointerstitial injury was defined as tubular damage, atrophy, interstitial inflammation, and fibrosis and was scored as previously described (34). Renal apoptosis was examined via the Click-iT Plus TUNEL Assay (Thermo Fisher Scientific, C10618). TUNEL^+^ nuclei were identified via fluorescence microscopy.

### Mitochondrial DNA copy number and qPCR of isolated TALs.

To quantify the relative content of mitochondrial DNA, total cellular DNA was extracted from the same amount of TAL (~4 cm) isolated from each group. Both mitochondrial (represented by the mitochondrial NADH-ubiquinone oxidoreductase chain 1 [*Mt-nd1*] gene) and nuclear (represented by the hexokinase 2 [*Hk2*] gene) DNA were evaluated under the same experimental conditions. The ratio of *Mt-nd1* to *Hk2* PCR products defines the relative mitochondrial DNA content. qPCR was conducted on a C1000 Touch Thermo Cycler (Bio-Rad). The protocol and primer sequences are detailed in the Supplemental Methods.

### TEM.

Clc-k2^–/–^ and WT kidneys were fixed in 2% paraformaldehyde/0.5% glutaraldehyde/2 mM calcium chloride (Electron Microscopy Sciences, 15732-10; Sigma, G5882; ACROS, 349615000) for 24 hours. The samples were postfixed with 2% glutaraldehyde and then 1% osmium tetroxide for 1 hour and rinsed with 0.1M sodium cacodylate buffer. After serial alcohol dehydration, the samples were embedded in Eponate 12 resin (Ted Pella, 18005). After the polymerization of Epon, the blocks were sectioned on an ultramicrotome, and ultrathin sections (70 nm) were poststained with uranyl acetate and lead citrate. The samples were examined via a Hitachi HT-7800 TEM.

### Seahorse cell mito stress test.

Cell mito stress tests using isolated TALs were conducted as previously described (Supplemental Methods) (17).

### Statistics.

Data analysis and curve fitting were performed using Prism (version 10.5.0) software (GraphPad Software). The data are presented as the mean ± SD. Statistical analysis was performed via Pearson’s test for correlations between the transcriptome and proteomics datasets; 2-way repeated-measures ANOVA with Geisser-Greenhouse correction followed by Šídák’s multiple-comparison test for 2-group comparisons over time or across cell cycle phases (CCK-8 assays and NPPB cell cycle experiments); 2-way repeated-measures ANOVA with Geisser-Greenhouse correction followed by Tukey’s multiple-comparison test for comparisons among 3 or more groups (cell cycle phase-specific differences among WT, Clc-k2^–/–^, and Clc-k2^–/–^/Wnk4^D561A/+^ TALs; growth curves; and water deprivation and DDAVP tests); 1-way ANOVA followed by Tukey’s multiple-comparison test for phenotype analyses and mitochondrial morphology analyses (mitochondrial volume and cristae densities) among multiple groups of mice; Mann-Whitney U test for cristae densities between 2 groups; and Welch’s 2-tailed *t* test for 2 groups. Survival curves for each group were compared using the log-rank test. Transcript-level counts were imported into R using the tximport Bioconductor package, and differential gene expression analysis was performed with DESeq2 (v1.46.0). For proteomic data, differential protein expression between Clc-k2^–/–^ and WT controls was evaluated using limma with a moderated *t* test, and *P* values were adjusted for multiple testing using the Benjamini-Hochberg procedure. Statistical significance was defined as a *P* value less than 0.05.

### Study approval.

All animal procedures were conducted in accordance with the University of Iowa’s guidelines for the care and use of laboratory animals. The IACUC at the University of Iowa approved this study (approval no. 4092421-003).

### Data availability.

A Supporting Data Values file is provided as supplemental material. The raw sequencing data and processed gene expression matrix have been deposited in the GEO under the accession no. GSE307980. The mass spectrometry proteomics data were submitted to ProteomeXchange under the accession no. PXD069389.

## Author contributions

CHH, YJC, and EM conducted the experiments, acquired the data, and analyzed the data. CCS analyzed the transcriptome data. CLH advised on the project, critically reviewed the data, and revised the manuscript. CJC designed the research studies, analyzed the data, created the figures, and drafted and finalized the paper. All authors approved the final version of the manuscript.

## Conflict of interest

The authors have declared that no conflict of interest exists.

## Funding support

This work is the result of NIH funding, in whole or in part, and is subject to the NIH Public Access Policy. Through acceptance of this federal funding, the NIH has been given a right to make the work publicly available in PubMed Central.

- NIH grants R01DK134420 (to CJC) and R01DK111542 (to CLH)

## Supplementary Material

Supplemental data

Supporting data values

## Figures and Tables

**Figure 1:**
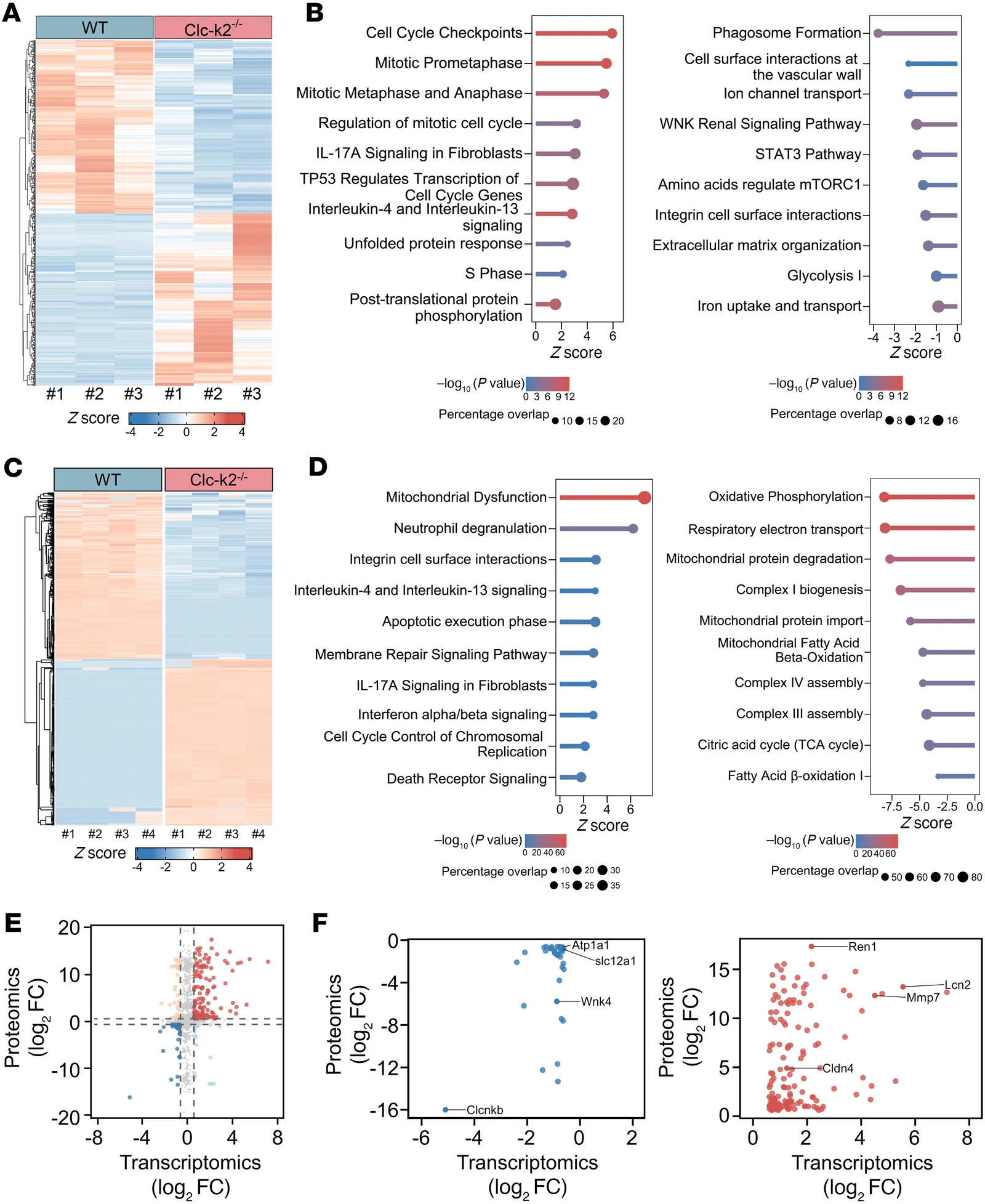
Transcriptomic and proteomic analyses of Clc-k2^–/–^ TALs and DCTs versus WT controls. (**A**) Heatmap displaying differential gene expression between Clc-k2^–/–^ and WT TALs microdissected from mouse kidneys (*n* = 3 per group). Differentially expressed genes are defined as those with *P* < 0.05 and absolute log_2_(fold change) > 0.6. Fold change (FC) is calculated by the ratio of expression levels between Clc-k2^–/–^ and WT groups. (**B**) Top pathways that are significantly upregulated (left) and downregulated (right) in Clc-k2^–/–^ TALs compared with WT controls. (**C**) Heatmap illustrating differential protein expression between Clc-k2^–/–^ and WT TALs and early DCTs isolated using anti-uromodulin antibody (*n* = 4 per group). Differentially expressed proteins are defined as those with *P* < 0.05 and absolute log_2_(FC) > 0.6. (**D**) Top pathways that are significantly upregulated (left) and downregulated (right) in Clc-k2^–/–^ TALs and DCTs compared with WT controls. (**E**) Correlation plot of the differentially regulated transcriptomics FC (*x*-axis) versus the proteomics FC (*y*-axis) (*r* = 0.58, *P* = 1.85 × 10^–14^) using Pearson correlation. (**F**) Synchronously regulated genes at both the transcriptomic and proteomic levels (left: 41 downregulated; right: 161 upregulated) are depicted, with selected hits highlighted.

**Figure 2:**
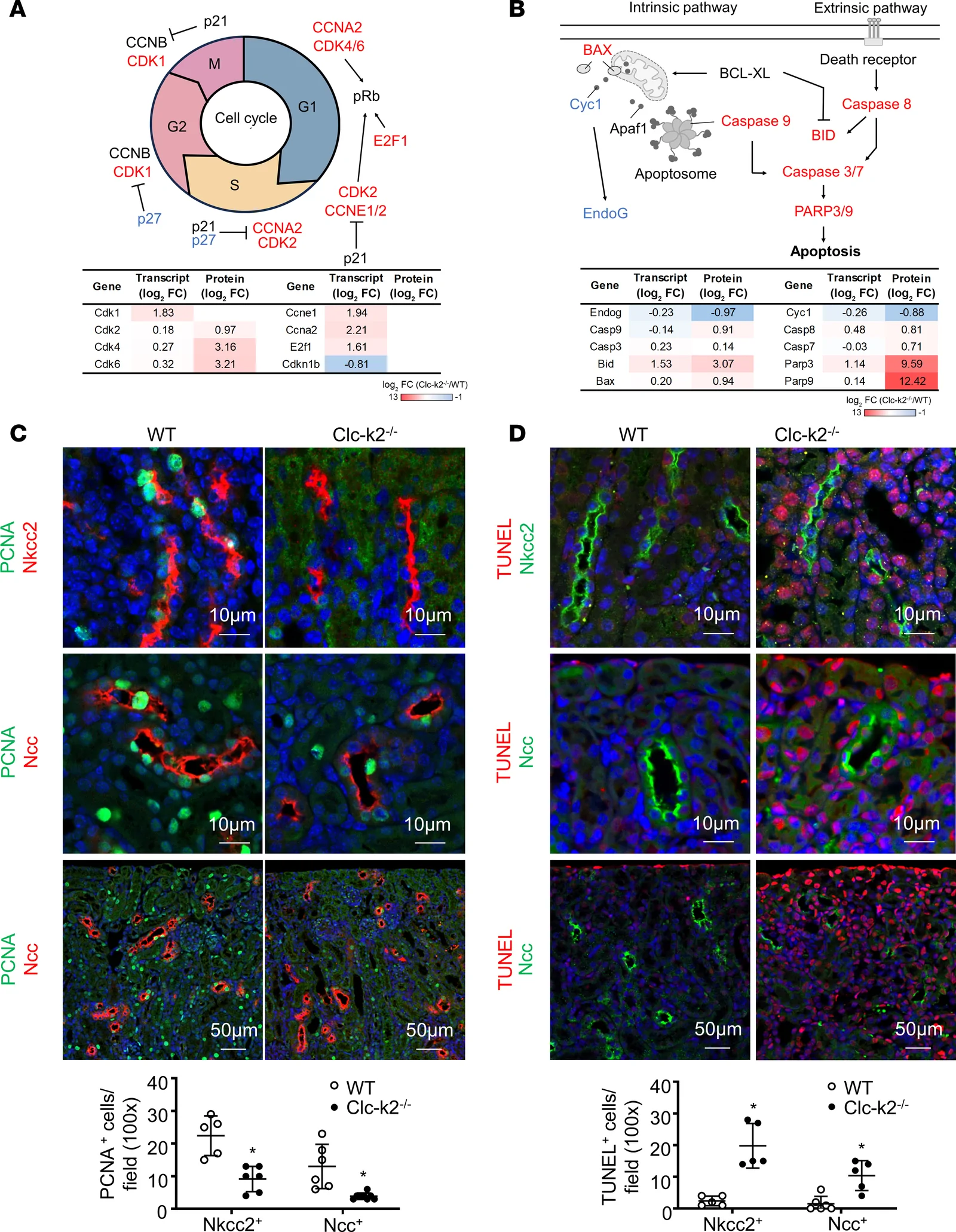
Clc-k2 affects cell proliferation, cell cycle progression, and apoptosis. (**A** and **B**) Schematic of the genes that regulate the cell cycle (**A**) or apoptosis (**B**). Genes that are significantly dysregulated are highlighted in red/blue font (red, upregulated; blue, downregulated), with relative fold changes (Log_2_FC: Clc-k2^–/–^ versus WT [WT]) in transcript (left) and protein (right) expression listed in boxes below. (**C**) Immunofluorescence costaining of the cell proliferation marker Pcna and the tubule-specific markers Nkcc2 (top, 400×) and Ncc (middle, 400×; bottom, 100×). The figures are representative of similar results (*n* ≥ 5). The number of proliferating TALs (both Pcna^+^ and Nkcc2^+^ cells) and DCTs (both Pcna^+^ and Ncc^+^ cells) per field (100×) was compared between Clc-k2^–/–^ and WT mice. *P < 0.05 using Welch’s *t* test. (**D**) Combined TUNEL assay and immunofluorescence staining of the tubule-specific markers Nkcc2 (top, 400×) and Ncc (middle, 400×; bottom, 100×). The figures are representative of similar results (*n* ≥ 5). The number of apoptotic TALs (both TUNEL^+^ and Nkcc2^+^ cells) and DCTs (both TUNEL^+^ and Ncc^+^ cells) per 100× field was compared between the Clc-k2^–/–^ and WT mice. *P < 0.05 using Welch’s *t* test.

**Figure 3:**
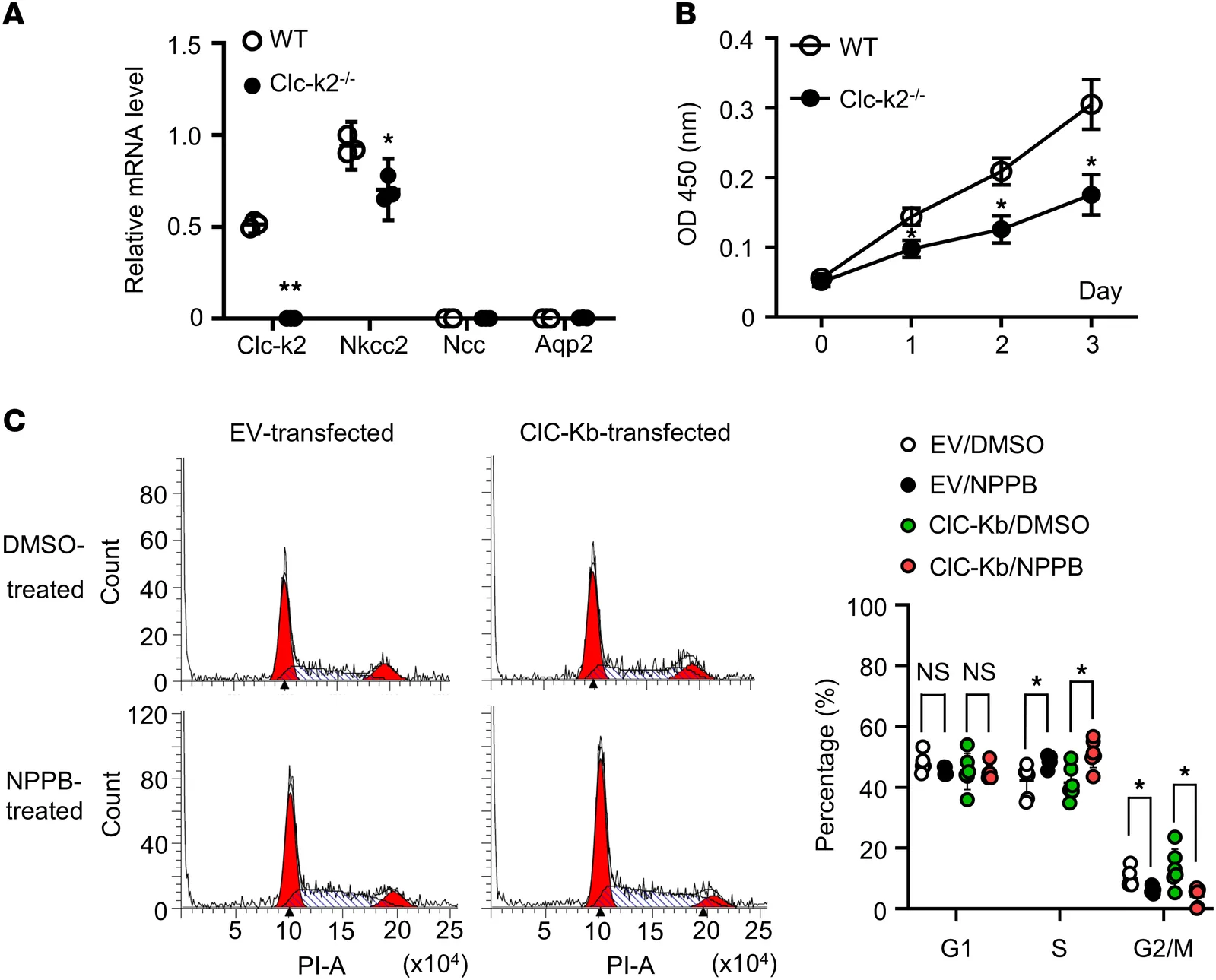
Clc-k2 deficiency or inhibition impairs cell proliferation and cell cycle progression. (**A**) The characteristics of primary-cultured TALs were validated by qPCR for Clc-k2 and tubule-specific markers (Nkcc2 for TALs, Ncc for DCTs, and Aqp2 for collecting ducts). The mRNA level of Nkcc2 in WT TALs was designated as 1 (*n* = 3, *P < 0.05, **P < 0.001 using Welch’s *t* test). (**B**) Comparison of the cell proliferation rate between primary-cultured Clc-k2^–/–^ and WT TALs using CCK-8 assays measuring optical density at 450 nm (OD 450) (*n* = 18, *P < 0.05 using 2-way repeated-measures ANOVA followed by Šídák’s multiple-comparison test). (**C**) Cell cycle analysis by propidium iodide staining of plasmid-transfected HEK-293 cells. Left: representative flow cytometric images of 6 similar results (*n* = 6). Right: the percentage of cells in each phase of the cell cycle from 6 experiments. *P < 0.05, between NPPB-treated and DMSO-treated HEK-293 cells using 2-way repeated-measures ANOVA followed by Šídák’s multiple-comparison test. All data are presented as mean ± SD. EV-transfected, empty vector plasmid-transfected HEK-293 cells; ClC-Kb–transfected, human ClC-Kb/Barttin plasmid-transfected HEK-293 cells.

**Figure 4:**
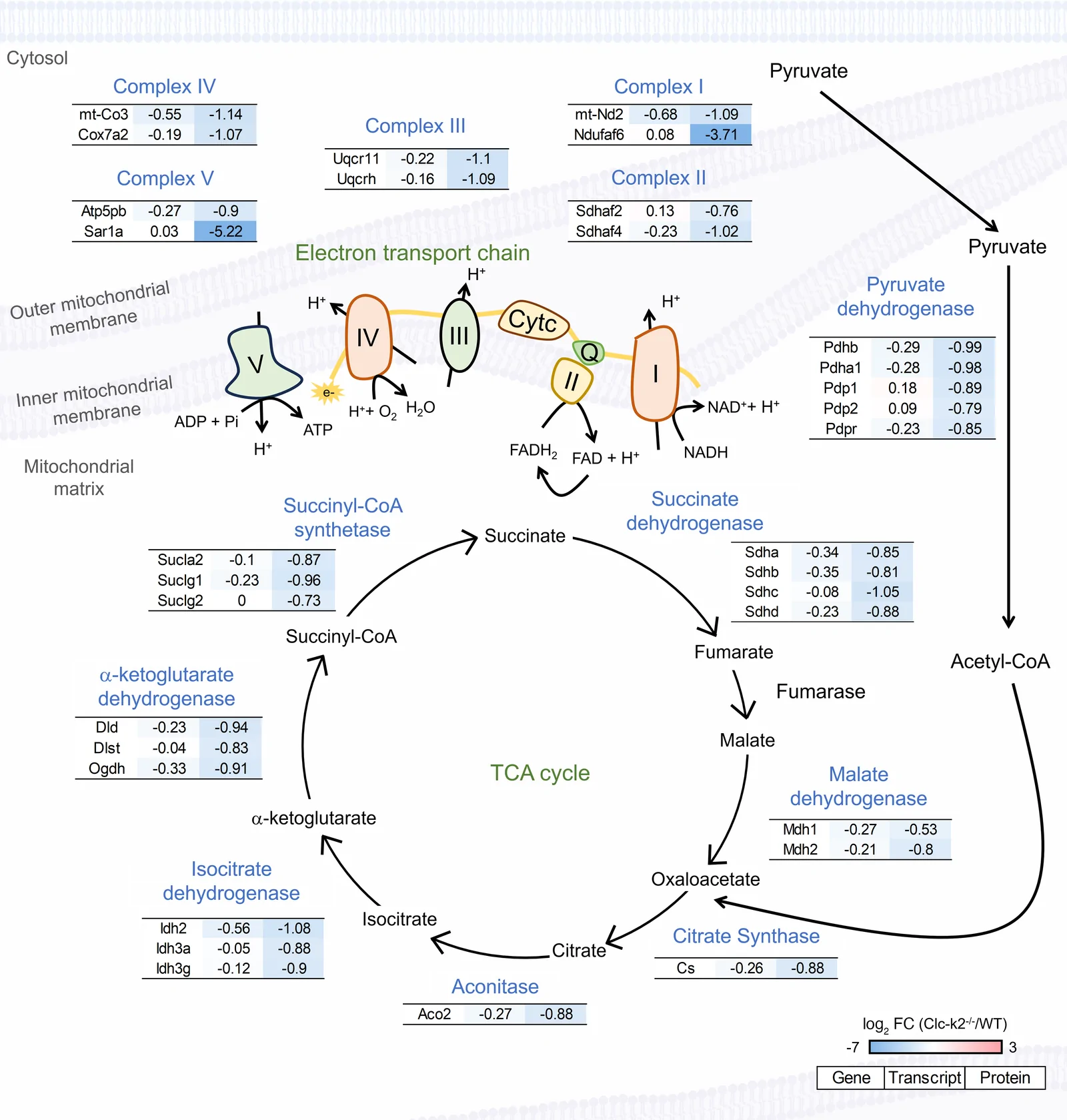
Differential regulation of the mitochondrial electron transport chain and the citric acid cycle in Clc-k2^–/–^ TALs and DCTs. Schematic of the proteins involved in mitochondrial oxidative phosphorylation. Proteins that are significantly downregulated are highlighted in blue font, with relative fold changes (log_2_FC: Clc-k2^–/–^ versus WT) in transcript (left) and protein (right) expression listed in boxes (red, upregulated; blue, downregulated). Cytc, cytochrome C; e^–^, high-energy electron; Pi, inorganic phosphate; Q, coenzyme q10.

**Figure 5:**
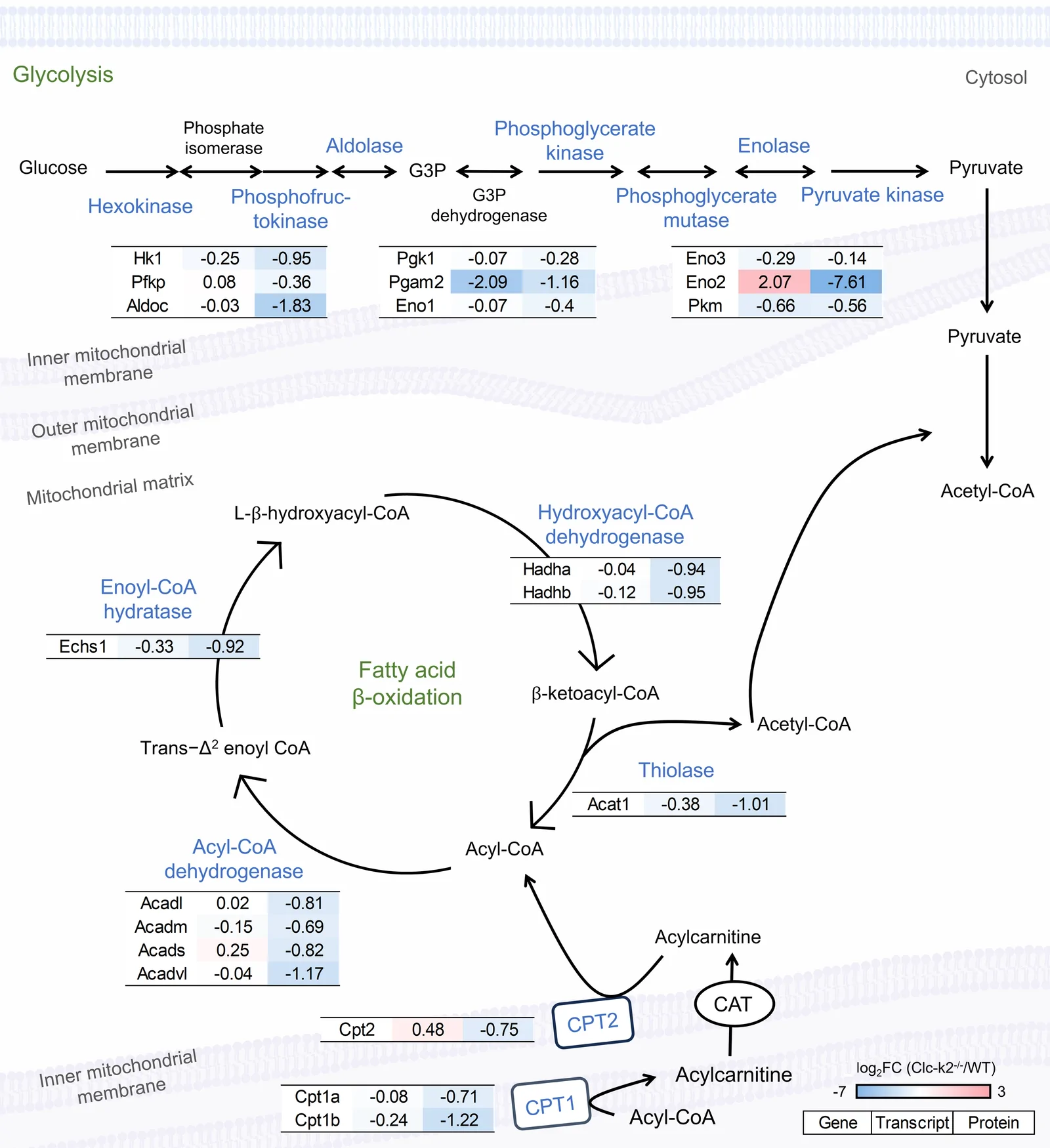
Differential regulation of glycolysis and fatty acid β-oxidation in Clc-k2^–/–^ TALs and DCTs. Schematic of the proteins involved in the pathways of glycolysis and fatty acid oxidation. Proteins that are significantly dysregulated are highlighted in blue (downregulated) or red (upregulated) font, with relative fold changes (log_2_FC: Clc-k2^–/–^ versus WT) in gene expression (left) and protein expression (right) listed in boxes (red, upregulated; blue, downregulated). CAT, carnitine acyltransferase; CPT1 and CPT2, Carnitine palmitoyltransferase I and II.

**Figure 6:**
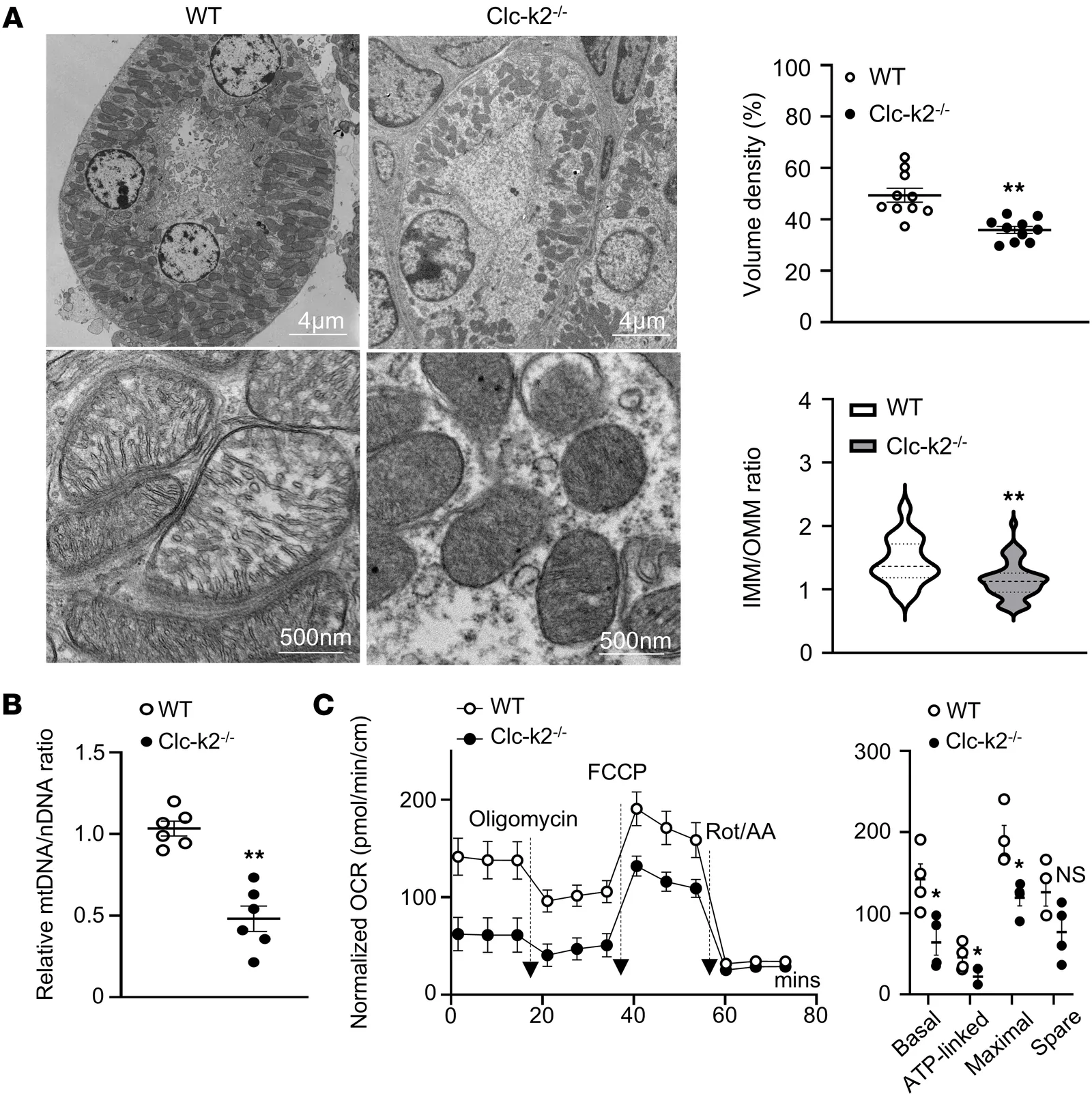
Reduced mitochondrial mass and respiration in Clc-k2^–/–^ TALs. (**A**) Left: representative TEM images of the TALs (top, 5,000×; bottom, 50,000×). Right: analyses of mitochondrial volume density (top) and cristae density (bottom). The mitochondrial volume density in TALs is defined as the percentage of the mitochondrial volume per renal tubular cell volume. Data from 10 tubules per group were compared using Welch’s *t* test (**P < 0.001). To quantify the mitochondrial crista density, the inner mitochondrial membrane-to-outer mitochondrial membrane (IMM/OMM) ratio was measured in 50 randomly selected mitochondria from 10 tubules in each group. The results were compared using the Mann-Whitney *U* test (**P < 0.001). All the TEM images were quantified using ImageJ (NIH). (**B**) The mitochondrial DNA-to-nuclear DNA ratio in isolated TALs relative to the WT average (*n* = 6) (**P < 0.001 using Welch’s *t* test). (**C**) Mitochondrial respiration in isolated TALs was assessed using Seahorse XF cell mito stress tests. The left panel displays the oxygen consumption rate (OCR) tracing from a representative test among 4 similar tests (*n* = 4). The dashed arrows indicate the sequential injections of oligomycin (mitochondrial ATPase inhibitor), FCCP (uncoupler), and rotenone/antimycin A (Rot/AA) (ETC complex I and III inhibitor). All OCRs were normalized to the tubule length. The right panel summarizes mitochondrial respiration parameters from 4 tests (*P < 0.05, using Welch’s *t* test). All data are presented as mean ± SD.

**Figure 7:**
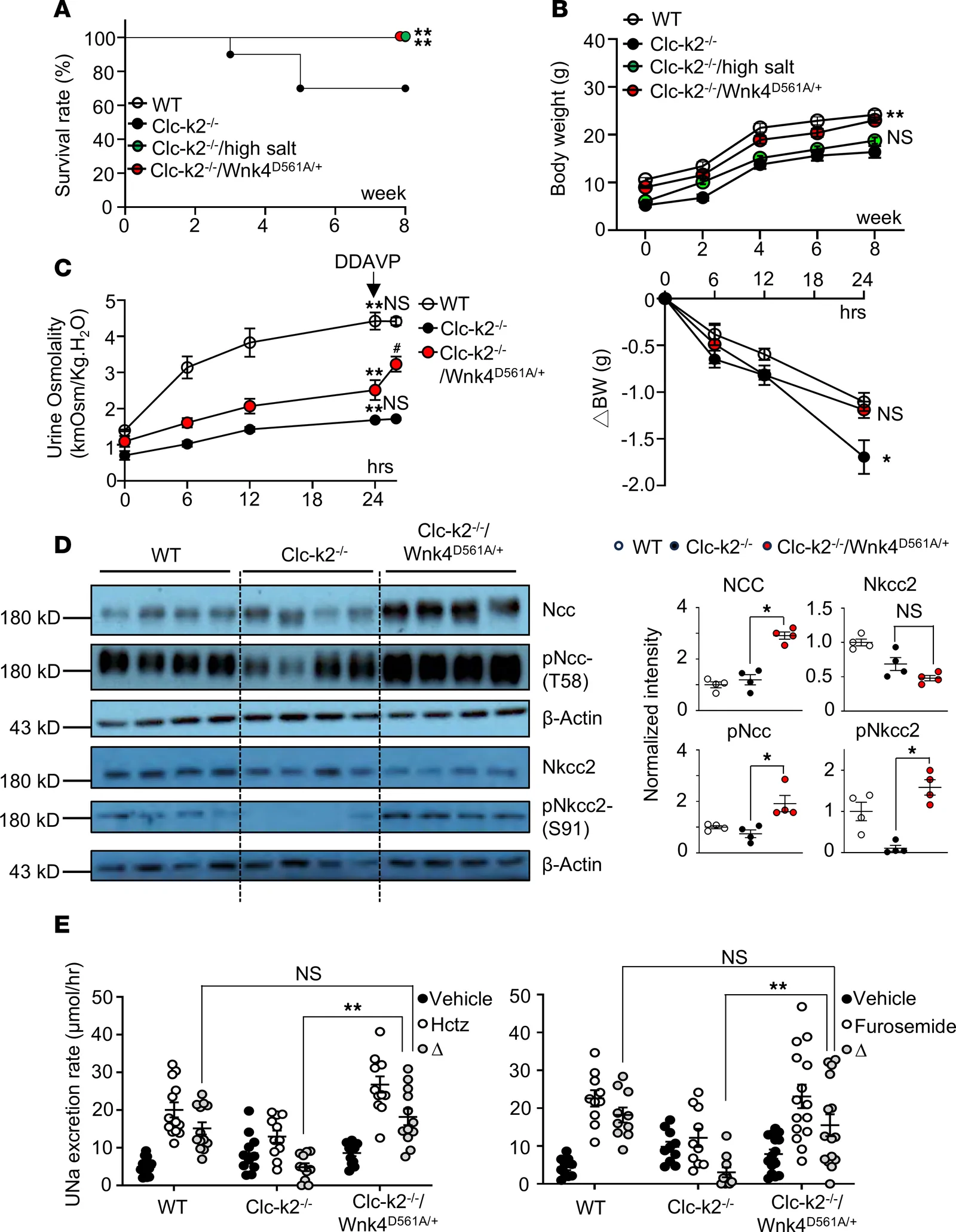
Gain-of-function Wnk4 mutation rescued Clc-k2^–/–^ mice. (**A** and **B**) Survival rates and growth curves of WT, Clc-k2^–/–^, Clc-k2^–/–^ on a high-salt diet (4% NaCl) (Clc-k2^–/–^/high salt), and Clc-k2^–/–^/Wnk4^D561A/+^ mice. The results were compared between the treatment group (Clc-k2^–/–^/high salt or Clc-k2^–/–^/Wnk4^D561A/+^) and the disease group (Clc-k2^–/–^) (*n* ≥ 10) (**P < 0.001, using the log-rank [Mantel-Cox] test for survival and 2-way repeated-measures ANOVA followed by Tukey’s multiple-comparison test for growth curves). (**C**) Serial measurements of urine osmolality (left) and body weight changes (right) during water deprivation and DDAVP injection (*n* ≥ 4). Comparisons were made for urine osmolality between the 0-hour and 24-hour marks during water deprivation (**P < 0.001) and between baseline and 2 hours after DDAVP injection (^#^*P* < 0.05, using 2-way repeated-measures ANOVA followed by Tukey’s multiple-comparison test). Net body weight changes after water deprivation were compared between the designated groups and the WT (^*^P < 0.05, using 2-way repeated-measures ANOVA followed by Tukey’s multiple-comparison test). (**D**) Left: Representative western blots of total and phosphorylated forms of Ncc and Nkcc2 from 4 independent experiments. Right: Densitometric analysis was performed using ImageJ, and values were compared between Clc-k2^–/–^ and Clc-k2^–/–^/Wnk4^D561A/+^ mice (*P < 0.05, using Welch’s *t* test). (**E**) The effects of hydrochlorothiazide (Hctz) or furosemide on diuretic-sensitive urine Na^+^ (UNa) excretion rates (Δ) were calculated and compared between the designated groups (*n* ≥ 10 per group, **P < 0.001, using Welch’s *t* test).

**Figure 8:**
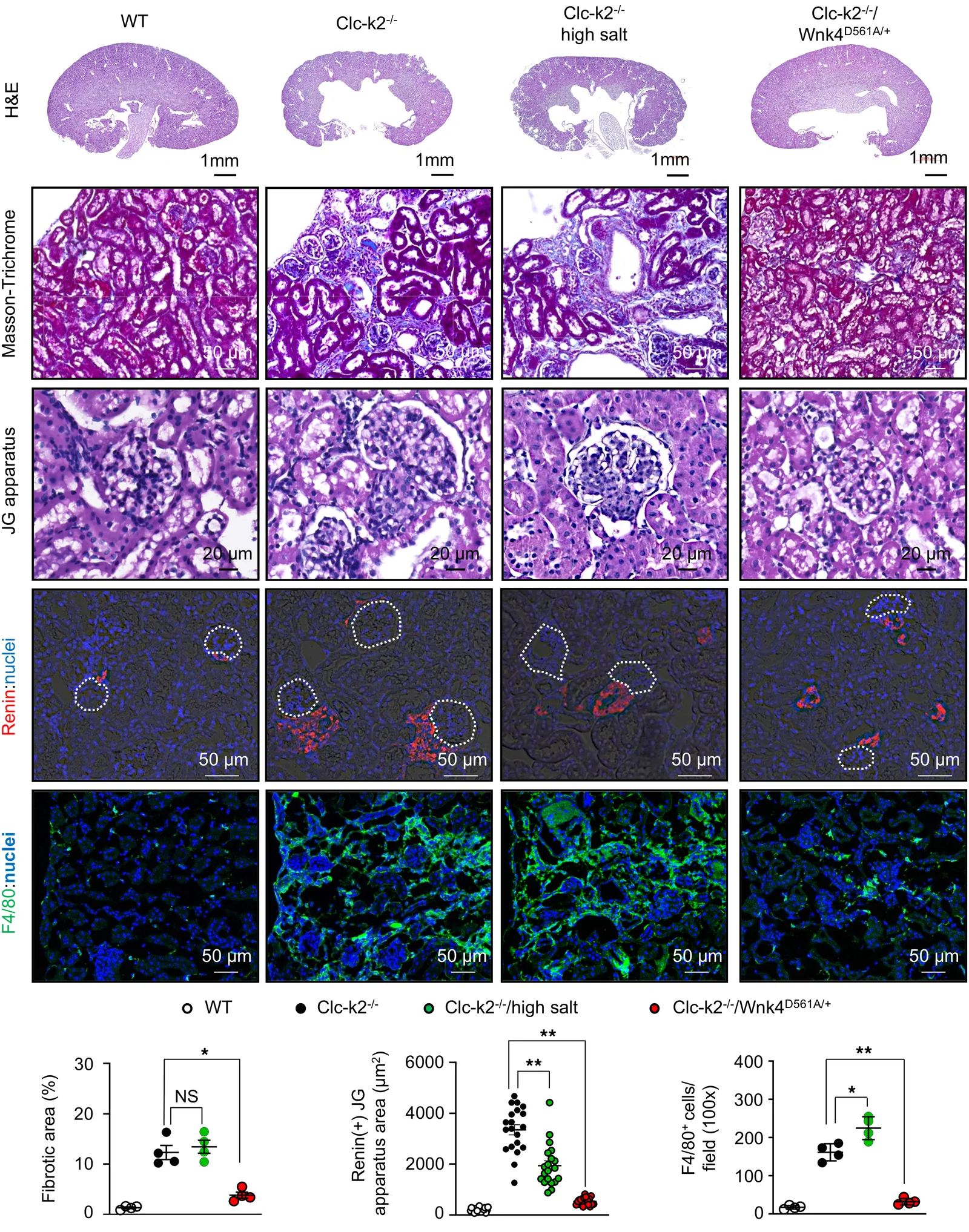
Wnk4^D561A/+^ rescues the renal histology of Clc-k2^–/–^ mice. The longitudinal kidney sections were stained with H&E, Masson trichrome, renin, or F4/80 to reveal the structure, interstitial fibrosis, renin-secreting cells, and macrophage infiltration, respectively. The dotted circles next to the renin-secreting juxtaglomerular (JG) cells indicate the parent glomerulus. The severity of interstitial fibrosis, JG apparatus hyperplasia, and macrophage infiltration was compared between the treatment group (Clc-k2^–/–^/high salt or Clc-k2^–/–^/Wnk4^D561A/+^) and the disease group (Clc-k2^–/–^) (*n* = 4, *P < 0.05, **P < 0.001) using Welch’s *t* test. Five random 100× fields were chosen from each sample for analysis. The results of the fibrotic area and the number of F4/80^+^ macrophages in 5 fields of a sample were averaged. The results for the JG apparatus size include all measurements from 20 selected 100× fields to reveal the size distribution.

**Figure 9:**
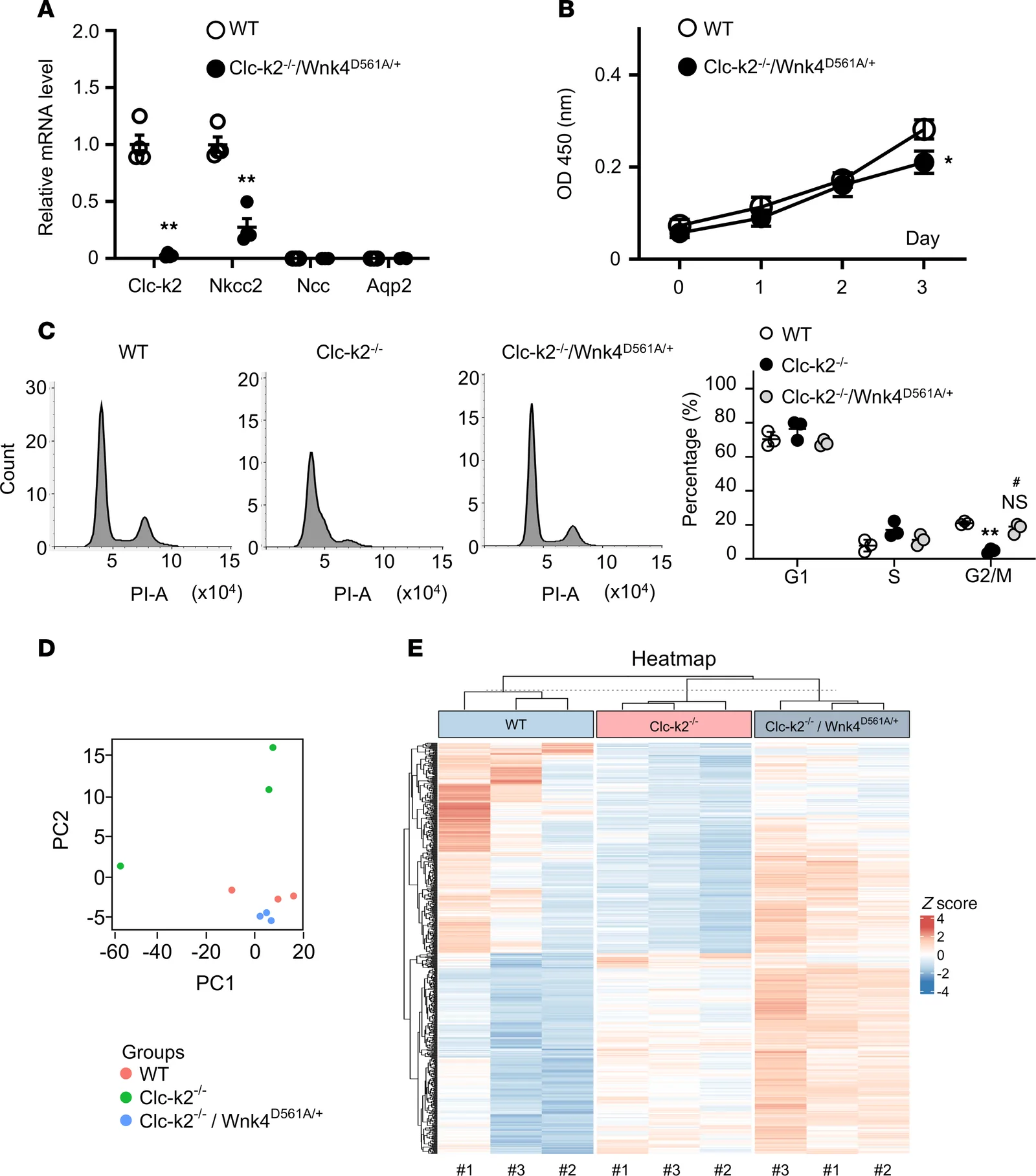
Wnk4^D561A/+^ rescues the cellular consequences of Clc-k2 deficiency. (**A**) The characteristics of primary-cultured TALs were validated by qPCR of Clc-k2 and tubule-specific gene expression. The mRNA level of Nkcc2 in WT TALs was designated as 1 (*n* = 4, **P < 0.001 using Welch’s *t* test). (**B**) Comparison of the cell proliferation rates between primary-cultured Clc-k2^–/–^/Wnk4^D561A/+^ TALs and WT TALs via CCK-8 assays (*n* = 8, *P < 0.05 using 2-way repeated-measures ANOVA followed by Šídák’s multiple-comparison test). (**C**) Cell cycle analysis of primary-cultured WT, Clc-k2^–/–^, and Clc-k2^–/–^/Wnk4^D561A/+^ TALs using propidium iodide (PI) staining. Left: representative flow cytometric images. Right: percentage of cells in each phase of the cell cycle. **P < 0.001 WT versus Clc-k2^–/–^; WT versus Clc-k2^–/–^/Wnk4^D561A/+^; and ^#^*P* < 0.05 Clc-k2^–/–^ versus Clc-k2^–/–^/Wnk4^D561A/+^ (*n* = 3, using 2-way repeated-measures ANOVA followed by Tukey’s multiple-comparison test). (**D**) Principal component analysis (PCA) reveals distinct gene expression profiles between experimental conditions. The first principal component (PC1) explains 77% of the total variance, while the second (PC2) accounts for 9%. Samples form clearly separated clusters in the 2-dimensional PCA space, indicating substantial transcriptional differences between conditions. (**E**) Heatmap of transcriptomic datasets from WT, Clc-k2^–/–^, and Clc-k2^–/–^/Wnk4^D561A/+^ TALs (*n* = 3 per group). Differentially expressed genes are defined as those with *P* < 0.05 and absolute log_2_(fold change) (Log_2_FC) > 0.6. All data are presented as mean ± SD.

**Figure 10:**
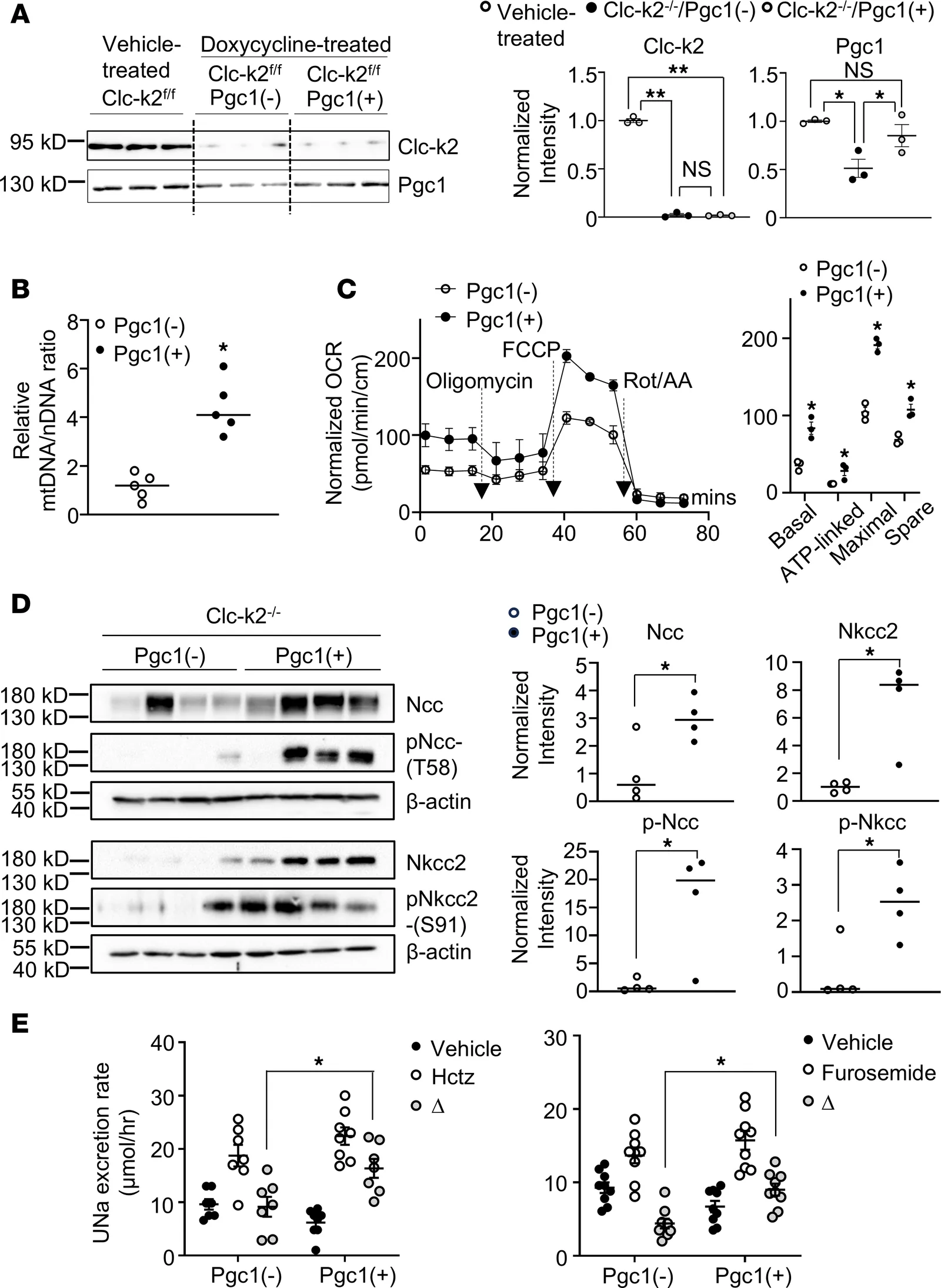
Pgc1α improves mitochondrial function and transepithelial salt transport in Clc-k2^–/–^ TALs and DCTs. (**A**) Western blots showing successful deletion of Clc-k2 and induction of the Pgc1 transgene. The density of the blots was analyzed by ImageJ and compared between the designated groups (*n* = 3 per group, *P < 0.05, **P < 0.001, using Welch’s *t* test). (**B**) The mitochondrial DNA-to-nuclear DNA ratio in isolated TALs relative to the average ratio in diseased TALs without Pgc1α transgene (*n* = 5 per group, *P < 0.05 using Welch’s *t* test). (**C**) Mitochondrial respiration in isolated TALs was assessed via Seahorse XF Cell Mito Stress Tests. The left panel displays the oxygen consumption rate (OCR) tracing from a representative test among 3 similar tests. The right panel summarizes mitochondrial respiration parameters from 3 tests (*n* = 3, *P < 0.05 using Welch’s *t* test). (**D**) Left: Representative Western blots of total and phosphorylated forms of Ncc and Nkcc2 from 4 independent experiments. Right: Densitometric analysis was performed using ImageJ, and values were compared between Clc-k2^–/–^ mice without [Pgc1(–)] or with [Pgc1(+)] the Pgc1α transgene (*P < 0.05 using Welch’s *t* test). (**E**) The effects of hydrochlorothiazide (Hctz) or furosemide on diuretic-sensitive urine Na^+^ (UNa) excretion rates (Δ) were calculated and compared between the designated groups (*n* ≥ 7 per group, *P < 0.05) using Welch’s *t* test. All data are presented as mean ± SD.

**Table 1:**
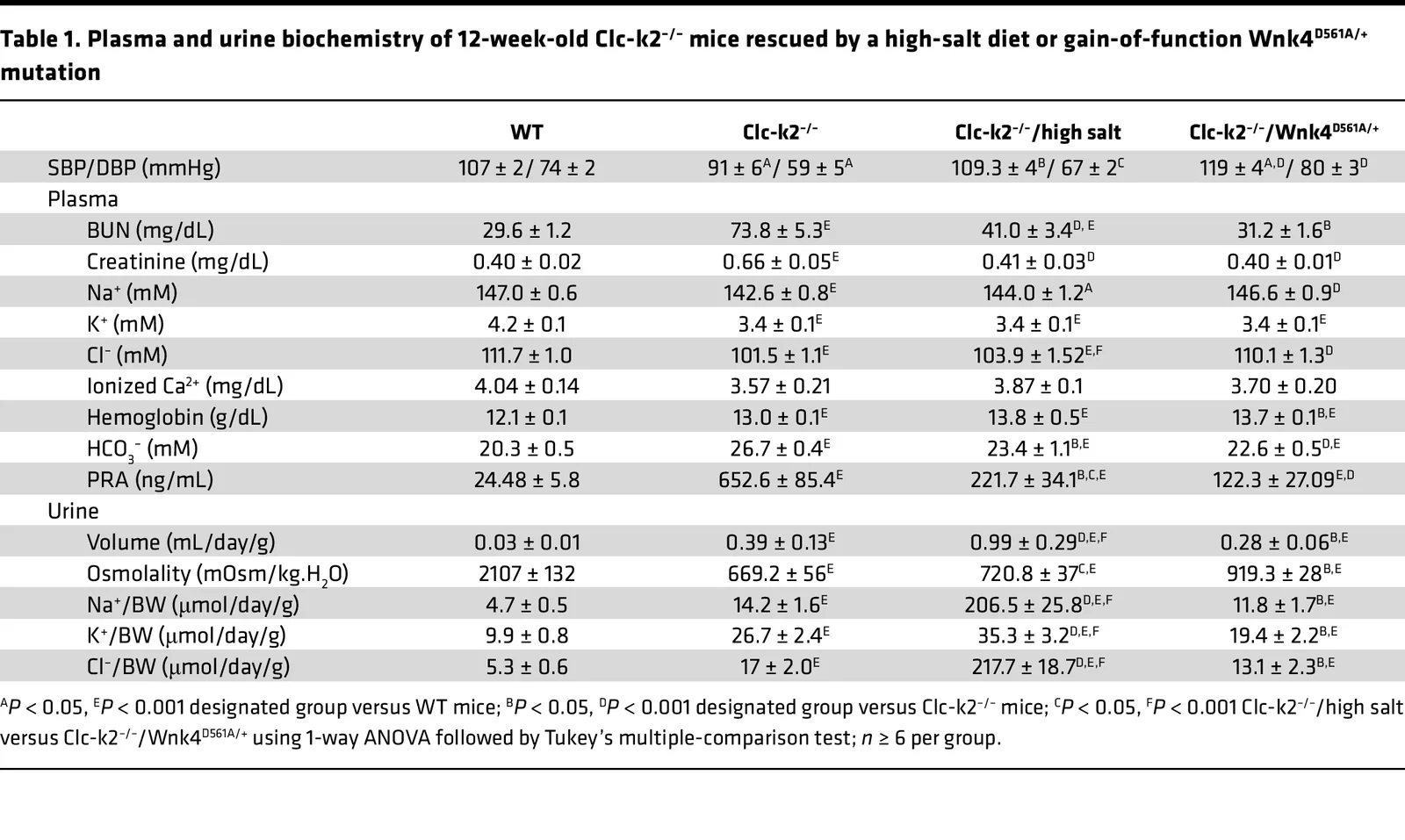
Plasma and urine biochemistry of 12-week-old Clc-k2^–/–^ mice rescued by a high-salt diet or gain-of-function Wnk4^D561A/+^ mutation

**Table 2:**
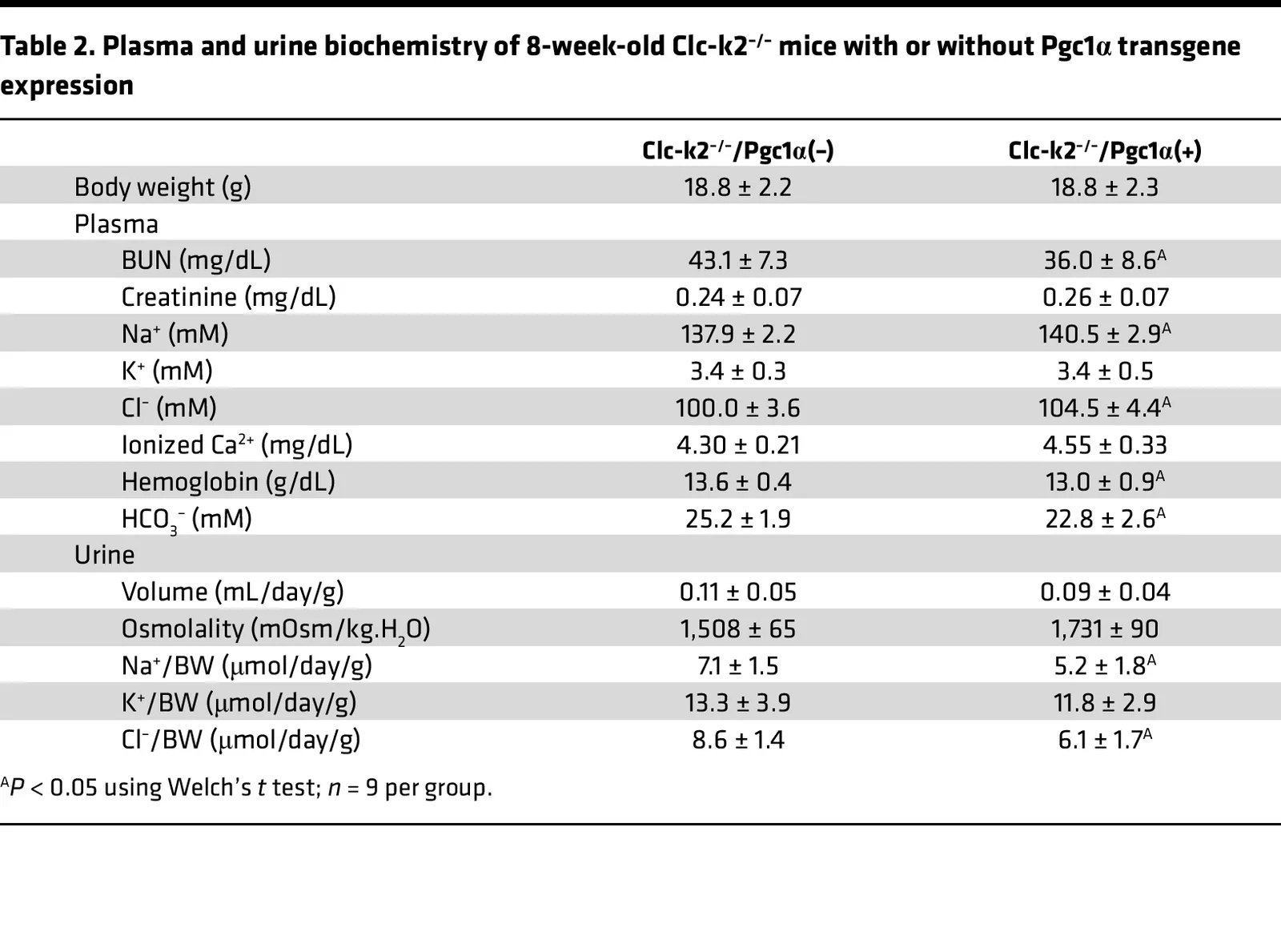
Plasma and urine biochemistry of 8-week-old Clc-k2^–/–^ mice with or without Pgc1α transgene expression

## Version 1 08/20/2026

In-Press Preview

## Version 2 10/08/2026

Electronic publication
