# Supplementary material for: Mitochondrial dysfunction underlies cell cycle arrest and tubular hypoplasia in ClC-Kb–deficient Bartter syndrome mice
Source: JCI Insight. 2026 Aug 20;11(19):e201067. doi: 10.1172/jci.insight.201067 (PMC13726409; doi:10.1172/jci.insight.201067)
Supplement: Supplementary file 1 [file jciinsight-11-201067-s219.pdf]

# Mitochondrial dysfunction underlies cell cycle arrest and tubular hypoplasia in ClC-Kb-deficient Bartter syndrome mice

Chiao-Hui Hsieh[1], Yu-Jen Chen[1, 2], Chih-Chien Sung[3], Emily Morrison[1], Chou-Long Huang[1], Chih-Jen Cheng[1, 3]

**SUPPLEMENTAL MATERIALS**

**Table of Contents:**

Supplemental methods
Supplemental Figure 1. Quality control of transcriptomic datasets.
Supplemental Figure 2. Validation of the characteristics of isolated TALs and DCTs.
Supplemental Figure 3. Quality control of proteomic datasets.
Supplemental Figure 4. Enhanced apoptosis in the proximal tubule and the collecting duct in Clc-k2$^{-/-}$ mice.
Supplemental Figure 5. Effects of $H_2O_2$ and furosemide on the cell cycle of HEK-293 cells.
Supplemental Figure 6. Wnk4D561A mutation improves mitochondrial morphology in Clc-k2$^{-/-}$ mice.
Supplemental Figure 7. Pgc1α improved mitochondrial biogenesis in inducible Clc-k2$^{-/-}$ mice.
Supplemental Figure 8. Pgc1α improved renal fibrosis in inducible Clc-k2$^{-/-}$ mice.
Supplemental Table 1. Differentially regulated genes that are commonly regulated at the mRNA and protein levels.
Supplemental Table 2. Differentially regulated genes that are differently regulated at the mRNA and protein levels.
Supplemental Table 3. Abbreviations used in this study.
Supplemental Table 4. Key resources.
References.

**Supplemental methods**

*Renal tubule microdissection.* Mouse kidneys were harvested, sliced into thin sections, incubated in a 15 mL tube with 10 mL of pre-warmed Hank's solution (composition (mM): 137 NaCl, 5 KCl, 0.8 $MgSO_4$, 0.33 $Na_2HPO_4$, 0.44 $KH_2PO_4$, 1 $MgCl_2$, 10 Tris(hydroxymethyl)amino methane hydrochloride, 0.25 $CaCl_2$, 2 glutamine, and 2 L-lactate, pH 7.4, osmolality 295-300 mOsm/kg.$H_2O$, bubbled with 95% $O_2$/5% $CO_2$) containing collagenase type I (1.5 mg/ml, Worthington) and 1% bovine serum albumin, and then gently shaken on a titer plate shaker at 37°C for 15-20 minutes. Then, the individual nephron segments were dissected in ice-cold Hank's solution pre-bubbled with 100% oxygen under a microscope. The isolated TALs were used for quantitative PCR, transcriptome analysis, primary cell culture, cell cycle and proliferation assays, DNA copy number analysis, and Seahorse assays.

*Purification of mouse primary TAL and DCT cells.* Mouse kidneys were minced and enzymatically digested in a digestion buffer containing 7.5 mg collagenase type I and 500 U DNase I (Roche) in serum-free DMEM/F12 medium (Thermo Fisher) at 37°C with constant shaking for 60 minutes. The enzymatic reaction was halted by adding DMEM/F12 supplemented with 10% FBS. The resulting cell suspension was passed through a 40 µm cell strainer (Fisherbrand) to obtain a single-cell suspension. For cell isolation, Dynabeads Biotin Binder (Thermo Fisher) were conjugated with biotinylated mouse anti-UMOD antibody (R&D Systems). After washing with PBS containing 1% BSA and 2 mM EDTA, the beads were incubated with the single-cell suspension at 4°C for 60 minutes. Labeled cells (UMOD-positive) were collected, washed, and lysed in 5% LDS diluted in Tris-HCl (pH 8.5).

*Transcriptome analysis.* Paired-end RNA sequencing reads were aligned to the mouse reference genome (mm10) using STAR aligner (v2.5.2b). Sequencing quality was assessed with FastQC. Transcript-level counts were obtained from BAM files using the featureCounts function of the Rsubread package (v2.8.2) with GENCODE vM23 gene annotations (https://www.gencodegenes.org/). Transcript-level counts were imported into R using the tximport Bioconductor package, and differential gene expression analysis was performed with DESeq2 (v1.46.0) (1). Normalized counts were extracted and transformed into z-scores for the selected gene sets. Data visualization included a heatmap generated using complexheatmap (v2.22.0) (2), and boxplots were created with ggplot2 (v3.5.2) (3). Pathway enrichment analysis was conducted using Ingenuity Pathway Analysis (IPA, version 01-23-01) on significantly differentially expressed genes ($Log_2$Fold change >0.6 or <– 0.6 and P-value < 0.05).

*Proteomic Profiling.* Peptide samples were analyzed on a timsTOF HT mass spectrometer (Bruker) coupled to a nanoElute2® UHPLC system (Bruker). Peptides were loaded onto a 25 cm × 150 µm ID analytical column packed with 1.5 µm C18 reverse-phase particles and separated using a linear gradient from 3% to 35% acetonitrile in 0.1% formic acid over 60 minutes at a flow rate of 500 nL/min. The column temperature was maintained at 50 °C. The mass spectrometer was operated in data-independent acquisition (DIA) mode using the diaPASEF method. Ion mobility separation was conducted over a range of $1/K_0$ = 0.7 to 1.3 Vs/cm², and precursor ions were fragmented across a mass range of m/z 300–1700 using a set of overlapping isolation windows. Each cycle comprised 8 diaPASEF scans, with a total cycle time of approximately 0.95 seconds.

The MS/MS spectral data were evaluated using PEAKS Studio (Version 13, Bioinformatics Solution Inc.). Data files were used to query the UniProt mouse database with the following parameters: precursor and fragment mass tolerances set to 20 ppm. Enzyme specificity was set to semi-specific, meaning one end can be non-tryptic. Fixed modification was Carbamidomethylation. Variable modifications included deamidation (NQ) and Oxidation (M). Proteins with more than one unique peptide were selected for further analysis in both animal models. It was assumed that the treatment-over-control protein ratios across all data were normally distributed. Processed proteomics data were analyzed using RStudio (2025.02.28) with R (version 4.4.3, February 2025). Data were generally handled with *tidyverse* (v2.0.0) (4). A linear model for differential expression (DE) analysis was generated in *limma* (v3.62.2) (5) with disease group and technical batch as covariates in the design model. PCA was performed with PCAtools (v2.8.0) (6). Comparisons between Clc-k2$^{-/-}$ and wild-type controls were performed in *limma* using a moderated t-test, adjusted by the Benjamini-Hochberg procedure. Pathway enrichment analysis was conducted using Ingenuity Pathway Analysis (IPA, version 01-23-01) on significantly differentially expressed genes ($Log_2$Fold change >0.6 or <–0.6 and P-value < 0.05). Figures were plotted with ggplot2 (v3.5.2) (3) or complexheatmap (v2.22.0) (2) when applicable.

*Western blot analysis, renal histology, and immunofluorescence staining*. For Western blotting, the protein concentration of the whole kidney lysate was measured, and 20 µg protein per sample was loaded onto an 8-10% SDS-PAGE gel. Proteins were separated by electrophoresis and transferred to a nitrocellulose membrane for Western blot analysis.

We raised the polyclonal anti-ClC-K antibody against peptide M1EELVGLREGASKKPVPL18 (7) and anti-NCC phospho-Thr-58 antibody against peptide F54GYN(pT)IDVVP63 (8) in rabbit and purchased the following antibodies: anti-NCC antibody (AB3553)(1:5000 dilution; Millipore), anti-NCC phospho-Thr58 antibody (1:5000 dilution), anti-NKCC2(AB2281)(1:2000 dilution; Millipore),anti-NKCC2 phospho-Ser-91 (S415C)(1:1000 dilution; Dundee), anti-PGC1 (ab54481)(1:2000 dilution; Abcam), anti-renin (ab212197)(1:200 dilution; Abcam), anti-PCNA (p8825)(1:500 dilution; Sigma), anti-F4/80 (ab6640)(1:200 dilution; Abcam). The secondary antibodies used in this study include anti-Rabbit (111-035-003)(1:4000 dilution; Jackson Lab), anti-Sheep IgG antibody (A130-101P)(1:10000 dilution; Bethyl), goat anti-rabbit Alexa Fluor Plus 555 (A-32732)(1:500 dilution; Invitrogen), donkey anti-rabbit Alexa Fluor 488 (A-21206)(1:500 dilution; Invitrogen), goat anti-rat Alexa Fluor 488 (Ab150157)(1:500 dilution; Abcam), goat anti-mouse Alexa Fluor 488 (115-545-003)(1:500 dilution; Jackson Lab). Nuclei were counterstained with 0.1 μg/ml DAPI.

*Quantitative PCR of isolated TALs.* Total RNA from isolated renal tubules was immediately extracted using RNeasy Plus Mini Kit (Qiagen, Venlo, Netherlands). Reverse transcription was performed using iScript™ Reverse Transcription Kit (Bio-Rad). We verified that no amplification was produced when reverse transcription was omitted from the sample. The relative mRNA abundance of genes of interest was standardized to glyceraldehyde-3-phosphate dehydrogenase (Gapdh) mRNA and calculated using ΔΔCt method. Each specimen was assayed in triplicate. Sequences of primers for mitochondrial-to-nuclear DNA ratio and RT-PCR analysis in this study are Mt-nd1, ctagcagaaacaaaccgggc (forward) and ccggctgcgtattctacgtt (reverse); HK2, gccagcctctcctgattttagtgt (forward) and gggaacacaaaagacctcttctgg (reverse); Sglt2, agaccttcgtcattcttgccg (forward) and gtgctggagatgttgccaacag (reverse); Nkcc2, ccagagcgttgtctaaagca (forward) and tgggcagctgtcatcactta (reverse); Ncc, gggtttgtgtcatgaggatg (forward) and ctcgtccgatcgtggtaga (reverse); Aqp2, ctggctgtcaatgctctccac (forward) and ttgtcactgcggcgctcatc (reverse); and Gapdh, cgtcccgtagacaaaatggt (forward) and tcaatgaaggggtcgttgat (reverse).

*Seahorse cell mito stress test.* Seahorse XFp analyzer was used for this study. The day before assays, the sensor cartridges were hydrated with calibrant and placed in a non-$CO_2$ incubator at 37°C overnight. On the day of the assay, Seahorse XFp miniplates were freshly coated with Cell-Tak solution (22.4 μg/ml). The assay medium (Seahorse XF DMEM with 10 mM glucose, 1 mM pyruvate, and 2 mM

glutamine or otherwise designated) and testing compounds were freshly prepared and pre-warmed in a 37°C bath following the manufacturer's protocol. Isolated TALs with total length ~1 cm were transferred to Cell-Tek-coated miniplates, centrifuged at 300 x g for 2 minutes, and incubated with 100% O2-bubbled assay medium in a non-$CO_2$ 37°C incubator for 1 hour prior to the assay. Isolated renal tubules were evenly distributed at the centers of the wells to ensure accurate measurements.

Next, the overnight-hydrated sensor cartridge was loaded with pre-warmed testing compounds and transferred to the XF analyzer for calibration. The injection sequence of testing compounds for cell mito stress test is: oligomycin A (inhibitor of mitochondrial ATPase, 3 μM) in port A, carbonyl cyanide-p-trifluoromethoxyphenylhydrazone (FCCP, uncoupler of OXPHOS, 1 μM) in port B, and rotenone/antimycin A (Rot/AA, inhibitor of mitochondrial complex I and III, 0.5 μM) in port C. Before starting the assay, the miniplate was removed from the incubator and carefully washed with fresh, pre-warmed assay media, without detaching the renal tubules. After the assays, the results were analyzed using Seahorse Analytics software (Agilent Technologies).

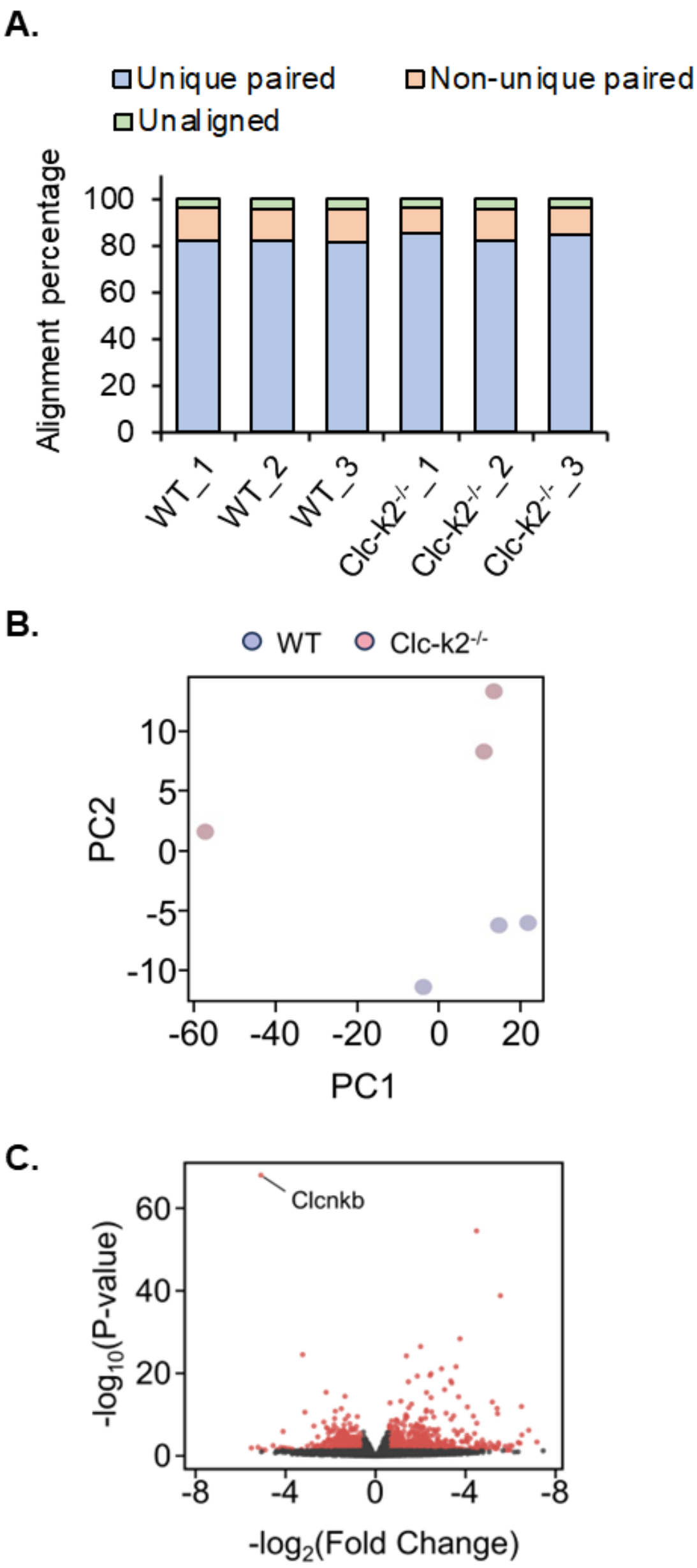


**Supplemental Figure 1. Quality control of transcriptomic datasets.** (**A**) The bar plot shows sequence alignment results, with each sample achieving at least 80% uniquely paired reads. (**B**) Principal component analysis (PCA) reveals distinct gene expression profiles between experimental conditions. The first principal component (PC1) explains 83% of the total variance, while the second (PC2) accounts for 9%. Samples form clearly separated clusters in the two-dimensional PCA space, indicating substantial transcriptional differences between conditions. (**C**) Volcano plots depicting the results of differential expression analyses from bulk RNA-sequencing. The x-axis represents the $\log_2$(fold change), while the y-axis shows the $-\log_{10}$(p-value). Each dot corresponds to a single gene (total genes: 41549). Red dots indicate significantly differentially expressed genes, defined by p-value $< 0.05$ and $|\log_2(\text{fold change})| > 0.6$. Black dots represent non-significant changes.

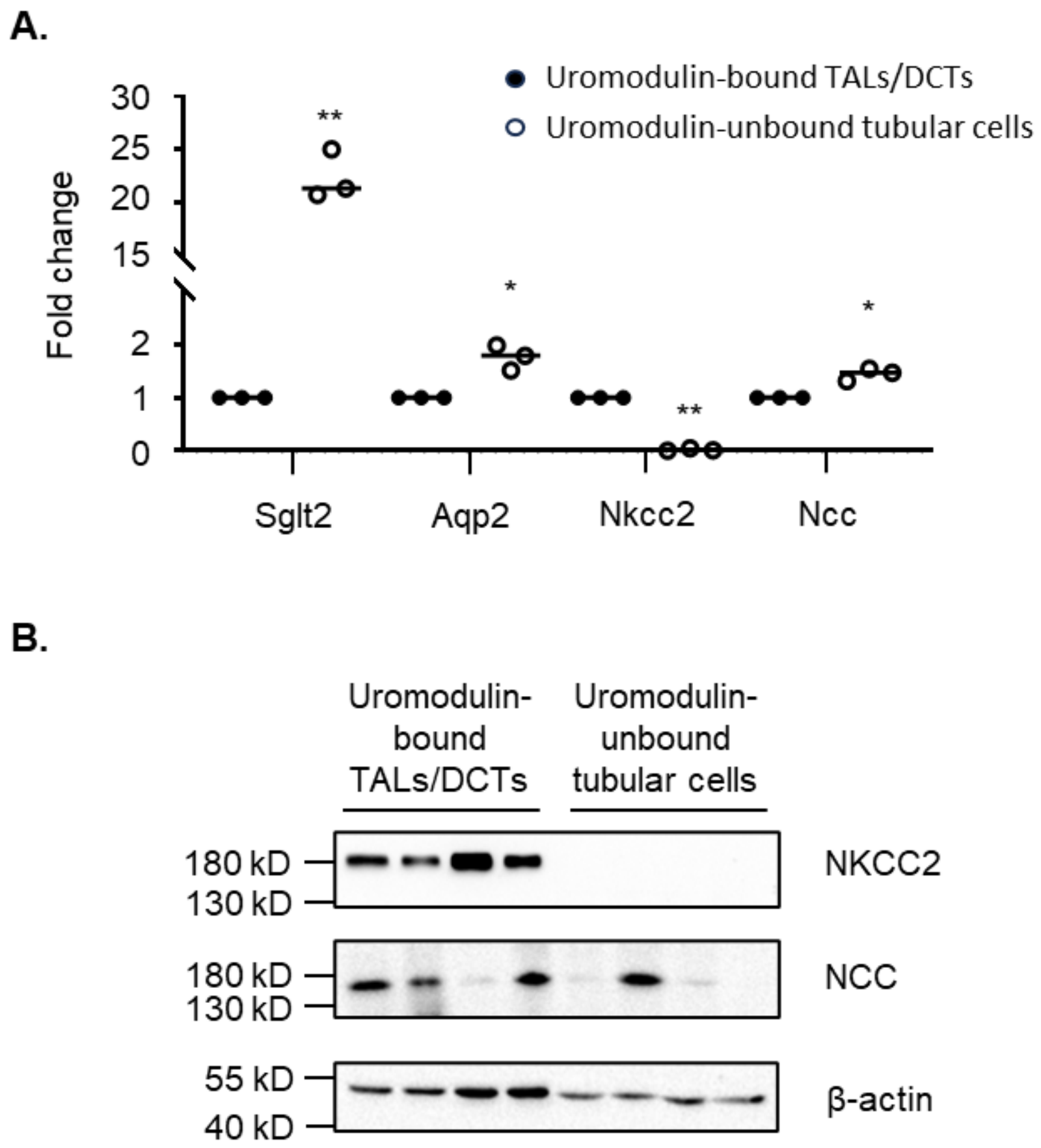


**Supplemental Figure 2. Validation of the characteristics of isolated TALs and DCTs.** (**A**) Quantitative PCR was applied to estimate the expression of segment-specific markers for proximal tubule (Sglt2), TAL (Nkcc2), DCT (Ncc), and collecting duct (Aqp2) in uromodulin-bound TALs/DCTs compared to those in uromodulin-unbound tubular cells (mainly proximal tubules). The expression level in uromodulin-bound TALs/DCTs is defined as 1. (**B**) Western blotting was performed to assess the expression of segment-specific markers, including Nkcc2 (TAL) and Ncc (DCT), in uromodulin-bound TALs/DCTs and uromodulin-unbound tubular cells. *$p$ < 0.05, **$p$ < 0.001, using Welch's t-test. All data are presented as means±SDs.

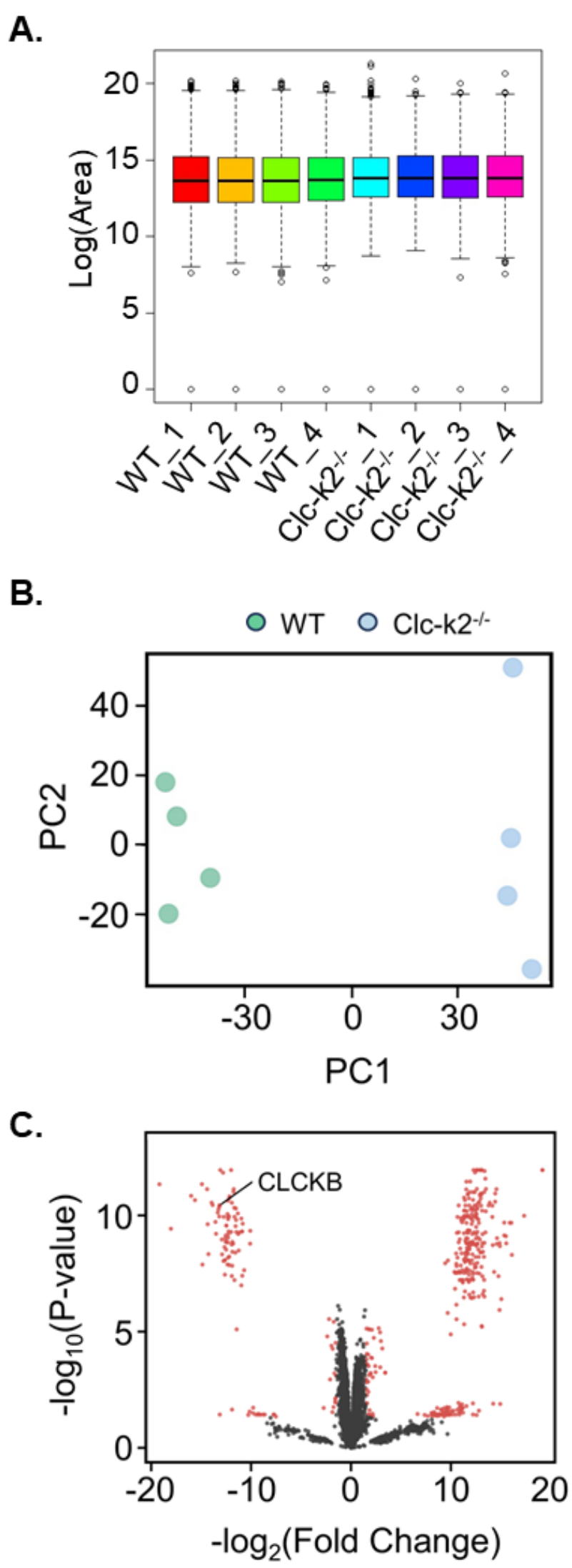


**Supplemental Figure 3. Quality control of proteomic datasets.** (**A**) Histogram plots of proteomics data after normalization are presented. Normalization was performed using a retention time (RT)-dependent total ion current (TIC) method to correct for systematic variations in signal intensity. The histogram shows similar distributions across samples, supporting data comparability. (**B**) Principal component analysis (PCA) reveals distinct gene expression profiles between experimental conditions. PC1 accounts for 49.9% of the total variance and PC2 for 13.3%. Samples form distinct clusters in the two-dimensional PCA space, highlighting substantial differences in proteomic profiles between the experimental groups. (**C**) Volcano plots depicting the results of differential expression analyses from proteomics data. The x-axis represents the $\log_2$(fold change), while the y-axis shows the $-\log_{10}$(p-value). Each dot corresponds to a single protein (total proteins: 5491). Red points indicate significantly differentially expressed proteins, defined by p-value $< 0.05$ and $|\log_2(\text{fold change})| > 0.6$. Black points represent non-significant changes.

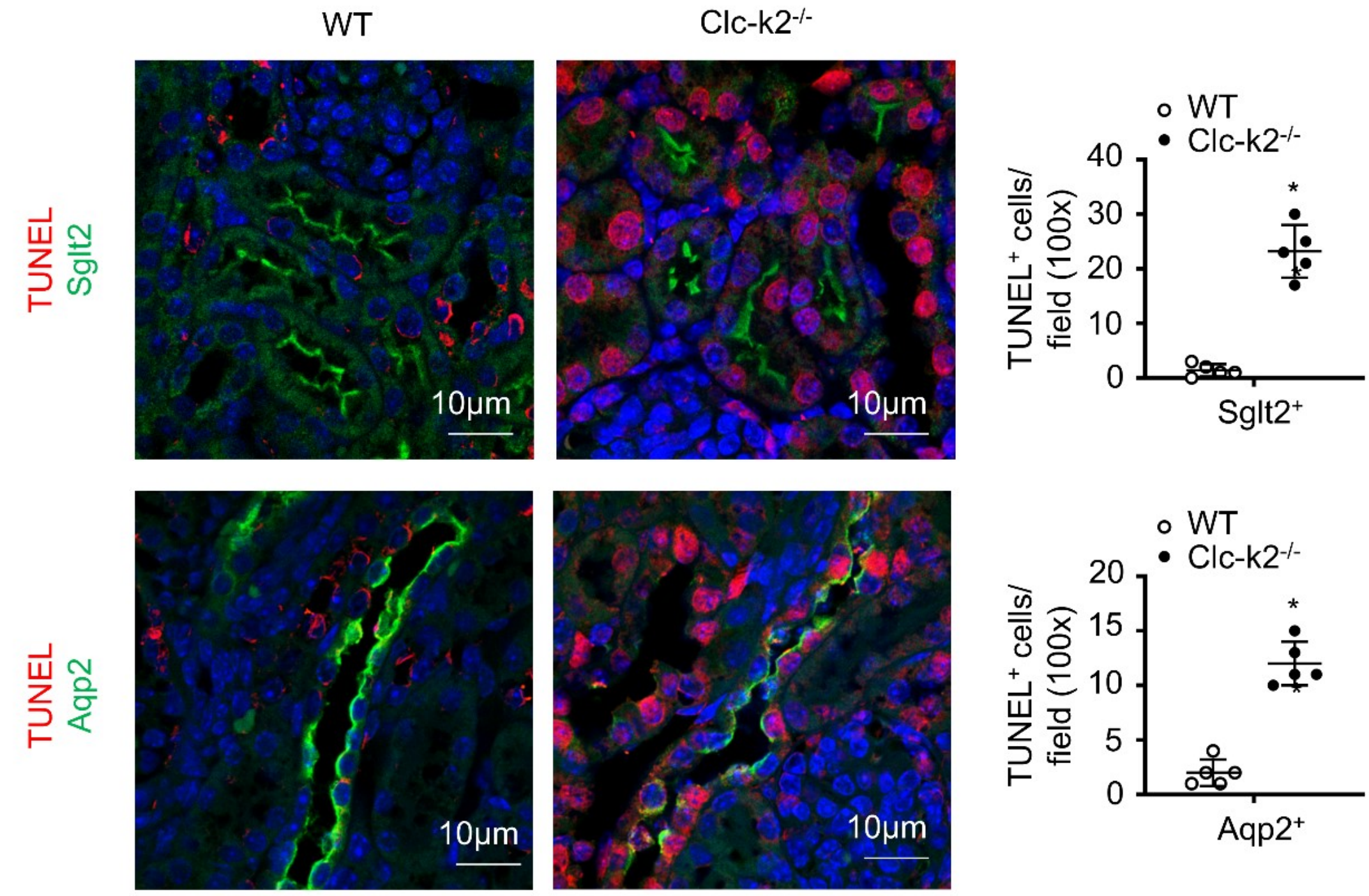


**Supplemental Figure 4**. **Enhanced apoptosis in the proximal tubule and the collecting duct in Clc-k2$^{-/-}$ mice.** Combined TUNEL assay and immunofluorescence staining of Sglt2 for the proximal tubule (top, 400x) or Aqp2 for the collecting duct (bottom, 400x). The number of apoptotic proximal tubular cells (both TUNEL- and Sglt2-positive cells) and collecting duct cells (both TUNEL- and Aqp2-positive cells) per field (100X) was compared between Clc-k2$^{-/-}$ and WT mice. The figures are representative of similar results (n≥5). *p<0.05 using Welch's t-test. All data are presented as means±SDs.

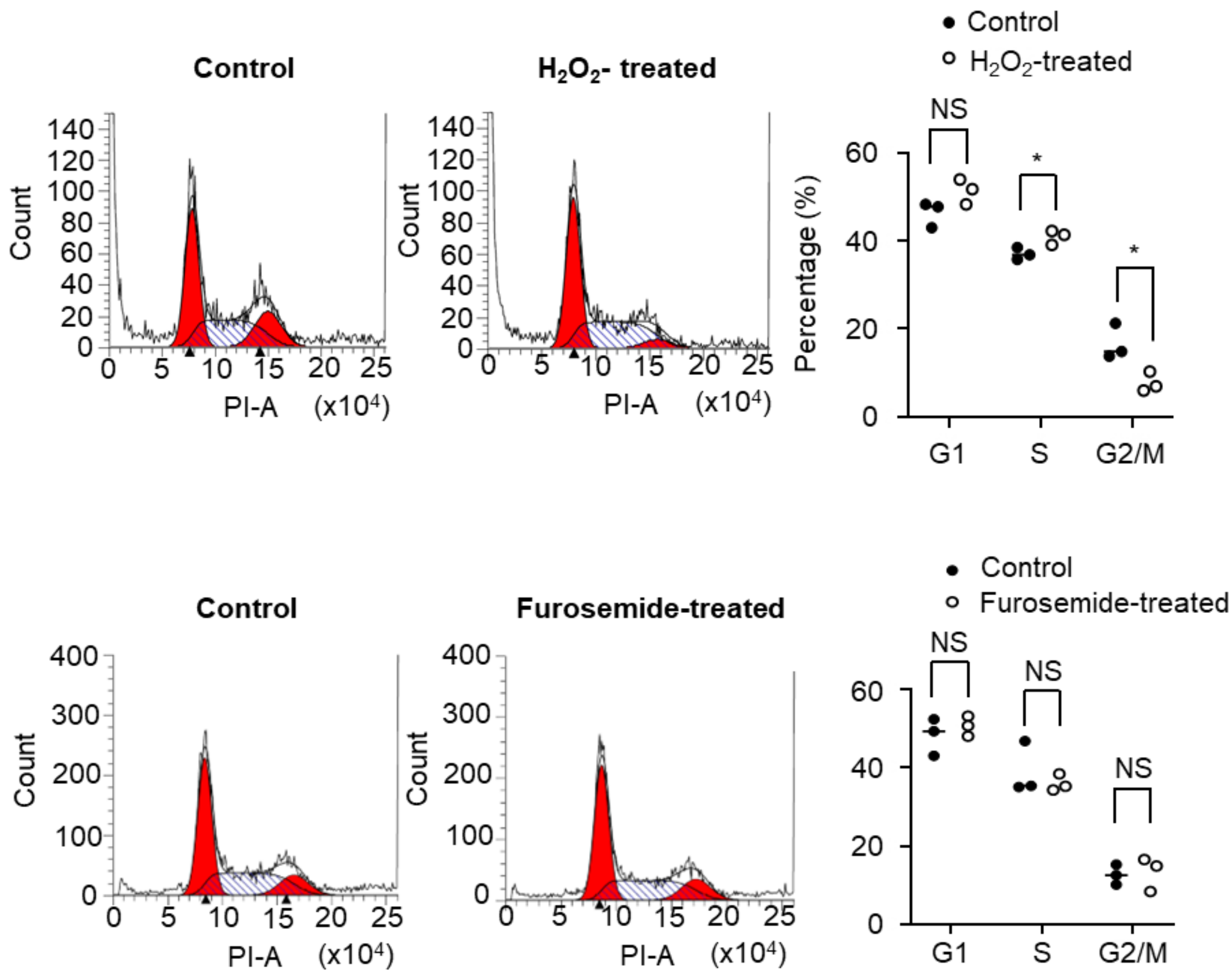


**Supplemental Figure 5. Effects of $H_2O_2$ and furosemide on the cell cycle of HEK-293 cells.** Cell cycle analysis by propidium iodide staining of HEK-293 cells treated with 0.2 M $H_2O_2$ or 100 μM furosemide versus vehicle (DMSO) for positive and negative control, respectively. Left: representative flow cytometry plots from three independent experiments (n = 3). Right: Quantification of the percentage of cells in each phase of the cell cycle from three independent experiments. $H_2O_2$ decreased cells in the G2/M phase (DMSO: 16.66 ± 4.02% vs. $H_2O_2$: 7.78 ± 2.27%, p = 0.03) and increased cells in the S phase (DMSO: 37.01 ± 1.36% vs. $H_2O_2$: 40.90 ± 1.62%, p = 0.03). *p < 0.05, NS, not significant, using two-way repeated measures ANOVA followed by Šídák's multiple comparisons test. All data are presented as means±SDs.

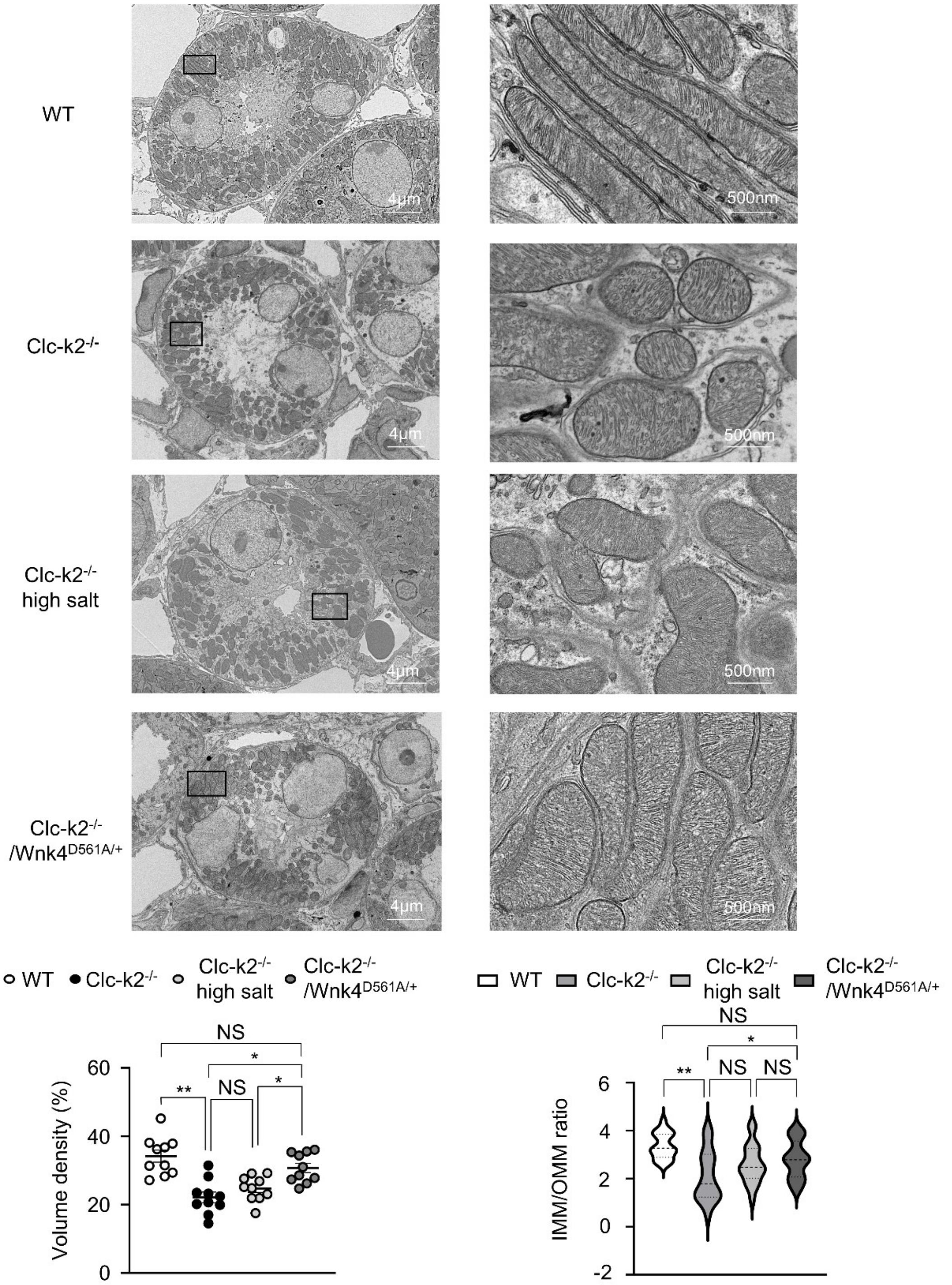


**Supplemental Figure 6**. **Wnk4D561A mutation improves mitochondrial morphology in Clc-k2$^{-/-}$ mice.** Top: left (5000x), right (50000x): representative TEM images of the TALs from wild-type, Clc-k2$^{-/-}$, Clc-k2$^{-/-}$ on a high-salt diet (4% NaCl), and Clc-k2$^{-/-}$/Wnk4$^{D561A/+}$ mice. Bottom: analyses of mitochondrial volume density (left) and cristae density (right). Mitochondrial volume density in TALs is defined as the percentage of mitochondrial volume per renal tubular cell volume. Data from ten tubules per group were compared. To quantify the mitochondrial crista density, the

inner mitochondrial membrane-to-outer mitochondrial membrane (IMM/OMM) ratio was measured in 20 randomly selected mitochondria from four tubules in each group. The results were compared using one-way ANOVA followed by Tukey's multiple comparisons test (**$p<0.001$, *$p<0.05$, NS: not significant). All TEM images were quantified using ImageJ (NIH). All data are presented as means±SDs.

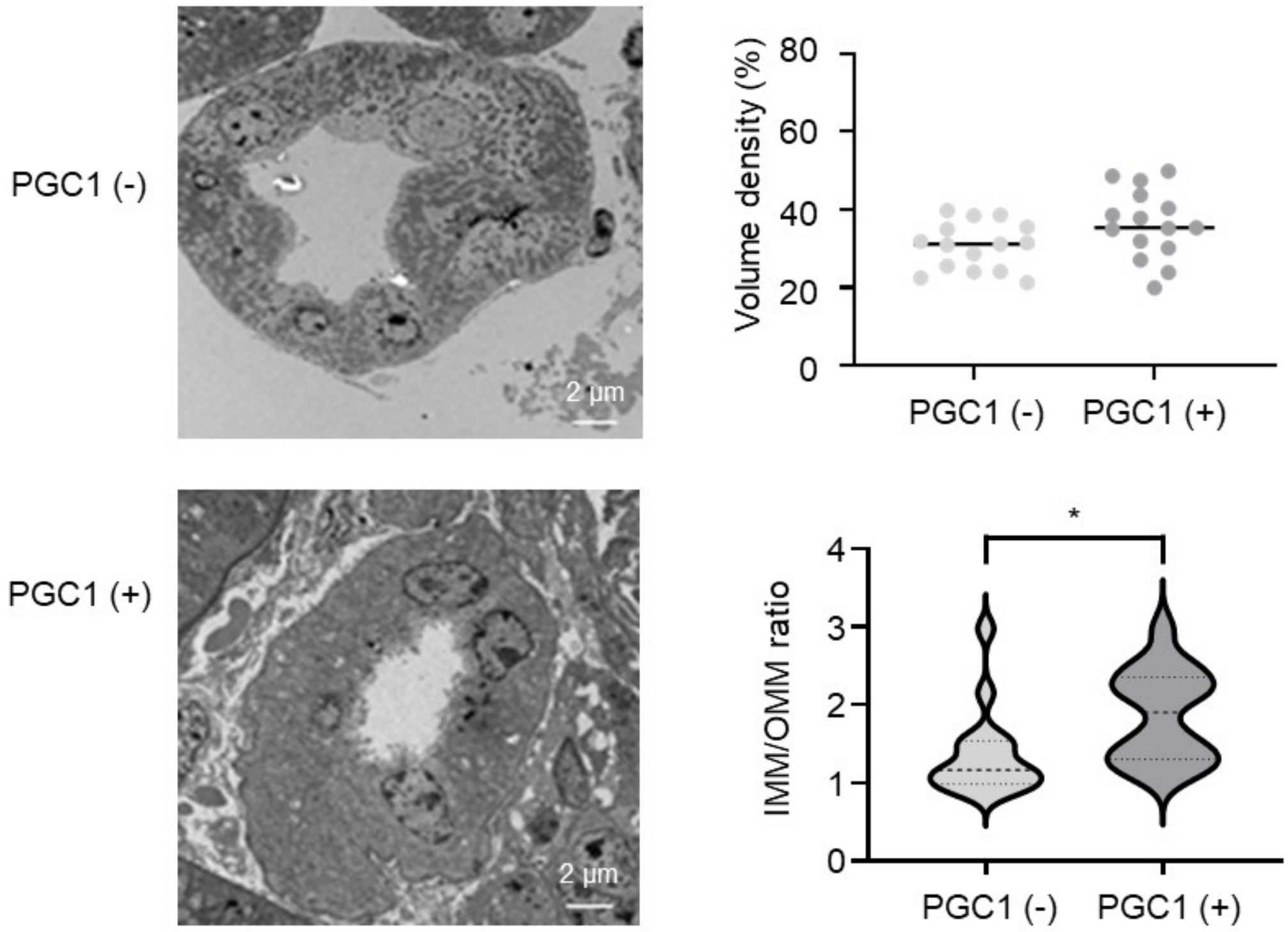


**Supplemental Figure 7**. **Pgc1α improved mitochondrial biogenesis in inducible Clc-k2$^{-/-}$ mice.** Left: representative TEM images of TALs from Clc-k2$^{-/-}$ without [Pgc1(-)] or with [Pgc1(+)] Pgc1α transgene expression. Right: analyses of mitochondrial volume density (top) and cristae density (bottom). The mitochondrial volume density in TALs is defined as the percentage of the mitochondrial volume per renal tubular cell volume. Data from 15 tubules per group were compared using Welch's t-test (*$p<0.05$). To quantify the mitochondrial crista density, the inner mitochondrial membrane-to-outer mitochondrial membrane (IMM/OMM) ratio was measured in 16 randomly selected mitochondria from 4 tubules in each group. The results were compared using the Mann-Whitney test (*$p<0.05$). All the TEM images were quantified using ImageJ (NIH). All data are presented as means±SDs. Pgc1α: peroxisome proliferator-activated receptor gamma coactivator 1α

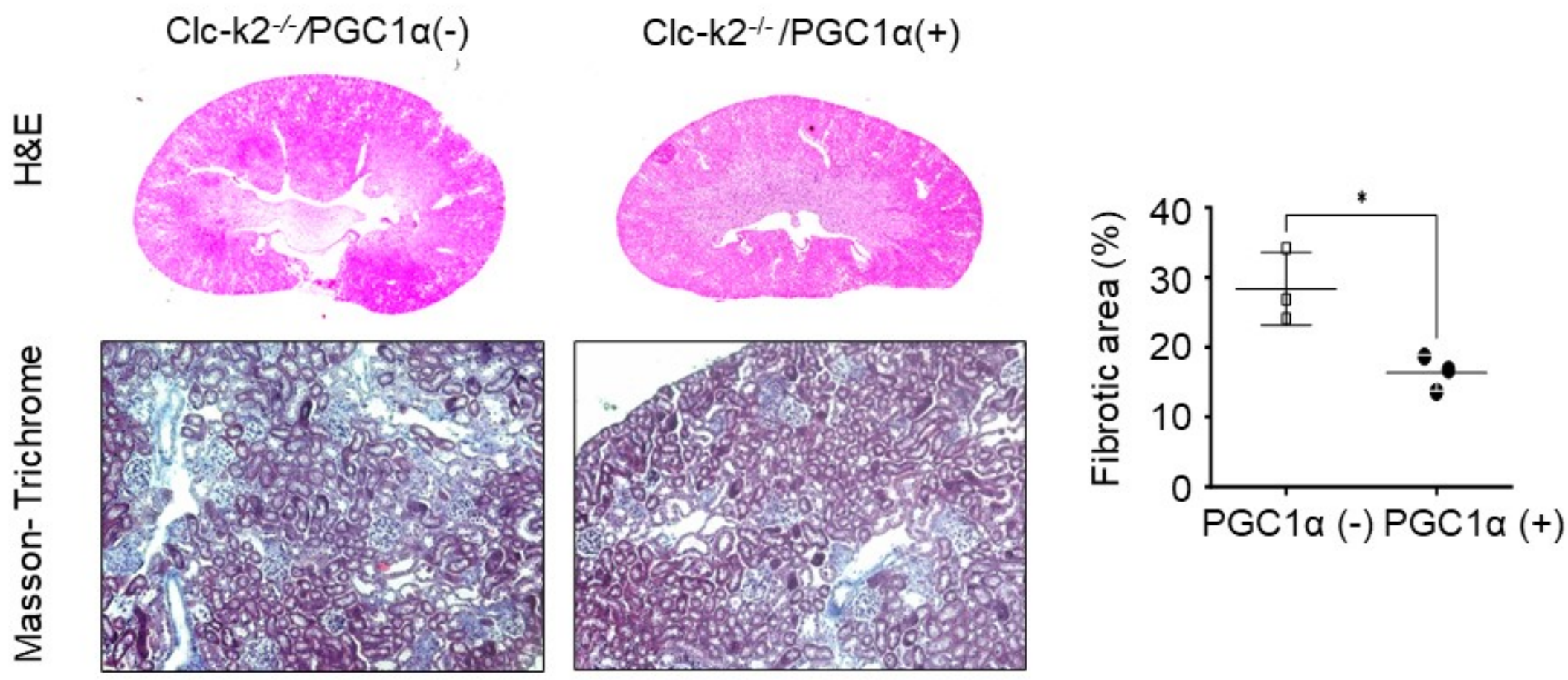


**Supplemental Figure 8**. **Pgc1α improved renal fibrosis in inducible Clc-k2$^{-/-}$ mice.** Kidneys harvested from 8-week-old mice were stained with H&E or Masson-Trichrome to assess the severity of interstitial fibrosis (n = 3 per group). In the statistical comparison (right), each dot represents the average of the fibrotic area in five selected fields of a sample. *$p<0.05$ using Welch's t-test. All data are presented as means±SDs.

**Supplemental Table 1.** Differentially regulated genes that are commonly regulated at the mRNA and protein levels. (see attached Excel file)

**Supplemental Table 2.** Differentially regulated genes that are differently regulated at the mRNA and protein levels. (see attached Excel file)

**Supplemental Table 3. Abbreviations used in this study**

| Abbreviation | Full name |
|---|---|
| BS | Bartter syndrome |
| TALs | Thick ascending limbs |
| DCTs | Distal convoluted tubules |
| $Clc\text{-}k2^{-/-}$ | Clc-k2(ClC-Kb mouse ortholog)-knockout |
| $Cl^-$ | Chloride |
| Wnk4 | With-no-lysine 4 |
| ETC | Electron transport chain |
| GOF | Gain-of-function |
| Pgc1α | Proliferator-activated receptor gamma coactivator 1-alpha |
| GEO | Gene Expression Omnibus |
| IL | Interleukin |
| TCA cycle | Tricarboxylic acid cycle |
| Kir | Inward-rectifying potassium channels |
| Cdk | Cyclin-dependent kinases |
| Casp | Caspases |
| Parp | poly (ADP–ribose) polymerases |
| Bax | BCL2-associated X protein |
| Bid | BH3 interacting-domain death agonist |
| CPT1 and 2 | Carnitine palmitoyltransferases 1 and 2 |
| NPPB | 5-nitro-2 (3 phenylpropylamino) benzoic acid |
| TEM | Trasnmission electric microscope |
| JG | Juxtaglomerular |
| Pgc1α-Tg | Pgc1α transgenic |
| LOF | Loss-of-function |
| DDAVP | 1-deamino-8-D-arginine vasopressin |
| FA | Formic acid |
| TEAB | Triethylammonium Bicarbonate |
| CAA | Chloroacetic acid |
| ACN | Acetonitrile |
| TFA | Trifluoroacetic acid |
| HEK293 | Human embryonic kidney cells |
| DMEM | Dulbecco's Modified Eagle Medium |
| PI | Propidium iodide |
| WB | Western blot |
| IF | Immunofluorescence |
| Mt-nd1 | Mitochondrial NADH-ubiquinone oxidoreductase chain 1 |
| Hk2 | Hexokinase 2 |
| IACUC | Institutional Animal Care and Use Committee |
| $Log_2FC$ | $log_2$(fold change) |
| CAT | Carnitine acyltransferase |
| Rot/AA | Rotenone/antimycin A |
| WT | Wild-type |

**Supplemental Table 3. Continued**

| Abbreviation | Full name |
|---|---|
| WT | Wild-type |
| OCR | Oxygen consumption rate |
| Hctz | Hydrochlorothiazide |
| H&E | Hematoxylin and eosin |
| PCA | Principal component analysis |
| PC | Principal component |
| UNa | Urine $Na^+$ |
| H&E | Hematoxylin and eosin |
| IPA | Ingenuity Pathway Analysis |
| DIA | Data-independent acquisition |
| DE | Differential expressio |
| Gapdh | Glyceraldehyde-3-phosphate dehydrogenase |
| FCCP | Carbonyl cyanide-p-trifluoromethoxyphenylhydrazone |
| RT-TIC | Retention time-dependent total ion current |

## Supplemental Table 4. Key resources

| Reagent type or resource | Designation | Source or reference | Identification |
|---|---|---|---|
| Consumable | S-Trap micro columns | ProtiFi LLC | C02-micro-80 |
| Consumable | C18 Spin Tips | Thermo Fisher | ZJ394337 |
| Consumable | Cell strainer | Fisherbrand | 22363547 |
| Consumable | Dynabeads Biotin Binder | Thermo Fisher | 11047 |
| Animal | Clcnkb-knockout (Clc-k2$^{-/-}$) mice | Our lab | ref. 9 |
| Animal | Wnk4$^{D561A/+}$ knock-in mice | Dr. Uchida's lab | ref. 10 |
| Animal | Tg(tetO-Ppargc1a)1Dpk/J (Pgc1αTg) mice | Jackson Laboratory | 12387 |
| Cell line | HEK-293 cells | ATCC | CRL-1573 |
| Reagent | RNeasy Plus Mini Kit | Qiagen | 74134 |
| Reagent | SMART-Seq mRNA LP | Takara Bio | 634769 |
| Reagent | AmPure XP magnetic beads | Beckman Coulter Genomics | A63880 |
| Reagent | High-Sensitive DNA kit | Agilent Genomics | 5067-4626 |
| Reagent | Qubit Protein assay Kit | Thermo Fisher | Q33212 |
| Reagent | Formaldehyde/glutaraldehyd | Electron Microscopy Sciences | 15949-60 |
| Reagent | Eponate 12 resin | Ted Pella | 18012 |
| Reagent | Cell Counting Kit-8 | Dojindo Molecular Technologies | CK04-01 |
| Reagent | Dulbecco's Modified Eagle Medium | Thermo Fisher | 11965092 |
| Reagent | Fetal bovine serum | Thermo Fisher | A56704-01 |
| Reagent | Lipofectamine 3000 | Thermo Fisher | L3000008 |
| Reagent | NPPB | Sigma-Aldrich | 107254-86-4 |
| Reagent | Propidium iodide | Thermo Fisher | A32959 |
| Reagent | Collagenase type I | Worthington | LS004197 |
| Reagent | DNase I | Roche | 10104159001 |
| Reagent | DMEM/F12 | Thermo Fisher | 11320033 |
| Reagent | iScript™ Reverse Transcription Kit | Bio-Rad | 1708890 |
| Plasmid | pCMV6-AC-GFP | ORIGENE | PS100010 |
| Plasmid | pCMV6-AC-CLCNKB-GFP | ORIGENE | RG228424 |
| Plasmid | Barttin | ORIGENE | RC213125 |
| Antibody | Biotinylated mouse anti-UMOD antibody | R&D Systems | BAF5175 |
| Antibody | anti-ClC-K antibody | peptide: M1EELVGLREGASKKPVPL | ref. 7 |
| Antibody | anti-NCC antibody | Millipore | AB3553 |
| Antibody | anti-NKCC2 antibody | Millipore | AB2281 |
| Antibody | anti-NCC phospho-Thr58 antibody | peptide F54GYN(pT)IDVVP63 | ref. 8 |
| Antibody | anti-NKCC2 phospho-Ser-91 | Dundee | S415C |
| Antibody | anti-NKCC2 phospho-Ser-130 | Dundee | S432C |
| Antibody | anti-PCNA antibody | Sigma | p8825 |
| Antibody | anti-F4/80 antibody | Abcam | ab6640 |
| Antibody | anti-Rabbit antibody | Jackson Lab | 111-035-003 |
| Antibody | anti-Sheep IgG antibody | Bethyl | A130-101P |
| Antibody | goat anti-rabbit Alexa Fluor Plus 555 | Invitrogen | A-32732 |
| Antibody | donkey anti-rabbit Alexa Fluor 488 | Invitrogen | A-21206 |
| Antibody | goat anti-rat Alexa Fluor 488 | Invitrogen | Ab150157 |
| Antibody | goat anti-mouse Alexa Fluor 488 | Jackson Lab | 115-545-003 |

**References**


1. Love MI, et al. Moderated estimation of fold change and dispersion for RNA-seq data with DESeq2. *Genome Biol.* 2014;15(12):550.
2. Gu Z, et al. Complex heatmaps reveal patterns and correlations in multidimensional genomic data. Bioinformatics. 2016;32(18):2847-2849.
3. Wickham H, et al. ggplot2: Create Elegant Data Visualisations Using Grammar of Graphics. https://cran.r-project.org/package=ggplot2. Accessed September 17, 2024.
4. Wickham H, et al. Welcome to the Tidyverse. *J Open Source Softw*. 2019;4(43):1686.
5. Ritchie ME, et al. limma powers differential expression analysis for RNA-sequencing and microarray studies. *Nucleic Acids Res.* 2015;43(7):e47.
6. Blighe K, Lun A. PCAtools: Everything Principal Components Analysis. https://github.com/kevinblighe/PCAtools. Accessed September 17, 2024.
7. Cheng CJ, et al. Functional severity of CLCNKB mutations correlates with phenotypes in patients with classic Bartter's syndrome. *J Physiol*. 2017;595(16):5573-5586.
8. Chen JC, et al. WNK4 kinase is a physiological intracellular chloride sensor. *Proc Natl Acad Sci U S A.* 2019;116(10):4502-4507.
9. Lin MH, et al. Impairment in renal medulla development underlies salt wasting in Clc-k2 channel deficiency. *JCI Insight*. 2021;6(20):e151039.
10. Yang SS, et al. Molecular pathogenesis of pseudohypoaldosteronism type II: generation and analysis of a Wnk4(D561A/+) knockin mouse model. *Cell Metab*. 2007;5(5):331-344.
